# Quantification Quality Control Emerges as a Crucial Factor to Enhance Single-Cell Proteomics Data Analysis

**DOI:** 10.1016/j.mcpro.2024.100768

**Published:** 2024-04-15

**Authors:** Sung-Huan Yu, Shiau-Ching Chen, Pei-Shan Wu, Pei-I Kuo, Ting-An Chen, Hsiang-Ying Lee, Miao-Hsia Lin

**Affiliations:** 1 Institute of Precision Medicine, College of Medicine, National Sun Yat-sen University, Kaohsiung, Taiwan; 2 School of Medicine, College of Medicine, National Sun Yat-sen University, Kaohsiung, Taiwan; 3 Department of Microbiology, National Taiwan University College of Medicine, Taipei, Taiwan; 4 Department of Urology, Kaohsiung Medical University Hospital, Kaohsiung, Taiwan; 5 Department of Urology, School of Medicine, College of Medicine, Kaohsiung Medical University, Kaohsiung, Taiwan

**Keywords:** single-cell proteomics, isobaric labeling, matching between runs, PSM-level normalization, differential expression analysis

## Abstract

Mass spectrometry (MS)-based single-cell proteomics (SCP) provides us the opportunity to unbiasedly explore biological variability within cells without the limitation of antibody availability. This field is rapidly developed with the main focuses on instrument advancement, sample preparation refinement, and signal boosting methods; however, the optimal data processing and analysis are rarely investigated which holds an arduous challenge because of the high proportion of missing values and batch effect. Here, we introduced a quantification quality control to intensify the identification of differentially expressed proteins (DEPs) by considering both within and across SCP data. Combining quantification quality control with isobaric matching between runs (IMBR) and PSM-level normalization, an additional 12% and 19% of proteins and peptides, with more than 90% of proteins/peptides containing valid values, were quantified. Clearly, quantification quality control was able to reduce quantification variations and q-values with the more apparent cell type separations. In addition, we found that PSM-level normalization performed similar to other protein-level normalizations but kept the original data profiles without the additional requirement of data manipulation. In proof of concept of our refined pipeline, six uniquely identified DEPs exhibiting varied fold-changes and playing critical roles for melanoma and monocyte functionalities were selected for validation using immunoblotting. Five out of six validated DEPs showed an identical trend with the SCP dataset, emphasizing the feasibility of combining the IMBR, cell quality control, and PSM-level normalization in SCP analysis, which is beneficial for future SCP studies.

## Highlights

• IMBR effectively expands the protein pools for DE analysis in multiplexed SCP.
• Removing cells and proteins with massive missing values improves cell separation.
• PSM normalization preserves the original data profile with efficient cell separation.
• Consistent trends in immunoblotting affirm this pipeline’s feasibility in SCP.

## Graphical Abstract

**Figure:**
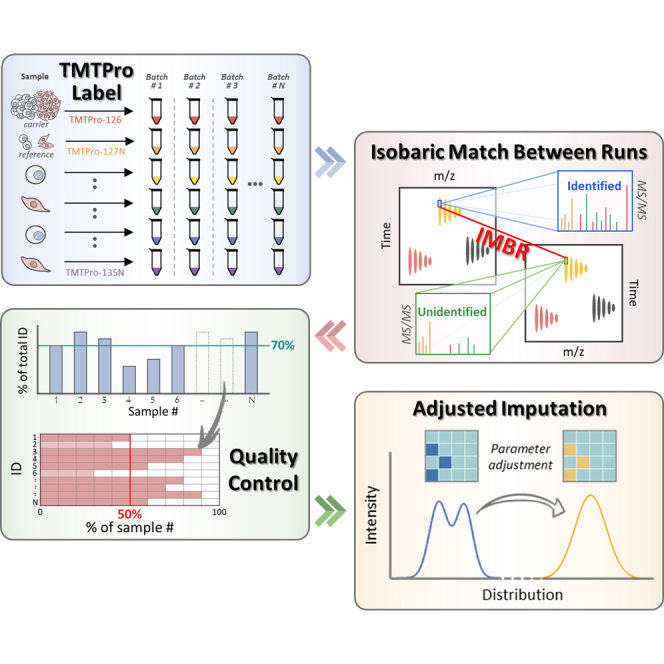

## In Brief

Single-cell proteomics (SCP) *via* mass spectrometry offers unbiased insight into cellular variability, overcoming antibody limitations. Despite instrument and sample prep advances, optimal data processing remains underexplored. Our approach, incorporating isobaric matching between runs, quantification quality control, PSMlevel normalization, and adjusted imputation, significantly improved SCP analysis. A 12 to 19% increase in quantified proteins and peptides, coupled with reduced variations and q-values, enhanced cell type separation. Immunoblotting validation of six DEPs affirmed the method's efficacy, highlighting its feasibility for future SCP studies.

Multicellular organisms rely on complex hierarchies of diverse cell types, working in concert to form specialized tissues that are necessary for carrying out multifarious physiological processes. The precise functional and morphological characteristics of each cell type are determined by a complex interplay between the genome, transcriptome, proteome, and other regulatory molecules. It is well known that different cellular states exhibit varying responses to identical stimuli, and even within the same cell type, the response can differ (1, 2). However, the traditional bulk methods, based on measuring collective dynamics, fail to reveal changes in cell population dynamics and cell-type-specific profiles which obscure the discovery of signaling networks underlying their biological phenotypes and cell-type-specific responses. For this reason, single-cell (SC) omics represents as a transformative technology to decipher pathologic insights, among which the genome and transcriptome take the central stage in this field due to the gene amplifiability and advances in next-generation sequencing (NGS). While gene signatures are of great value, it is difficult to predict the proteome behaviors, which are effector molecules of cells, since the discordance between mRNA and protein abundances (3, 4, 5) and the crucial roles of protein post-translational modifications (6).

Finding novel biological regulators ultimately relies on protein-centric approaches, making a rising technique demand for SCP analysis that allows direct identification and quantification, and provides molecular insights into cell states. Currently, antibody-based detection, such as CyTOF and SC barcode chips, is the most widely used approach to quantify targeted proteins at the SC level (7). Although this approach enables processing thousands of cells, it is extensively dependent on the availability of high-quality antibodies, limiting its utility in hypothesis-free, global data-driven biological interrogations (8). Instead, MS-based proteomics presents as a non-targeted method to identify and quantify proteins across individual cells (9). However, the challenges are the instrument sensitivity as well as the faithful transfer of every protein content of SC into MS in sample preparation (10). The innovations of nanoscale devices such as proteoCHIP (11), nanoPOTS (12), and nPOP (13) together with the additive of MS-compatible surfactants in reaction tubes all significantly reduce sample loss by preventing peptide surface absorption (14, 15). In 2018, SCoPE-MS (16), a multiplexing approach involved labeling the peptides with isobaric tandem mass tags (TMT) alongside a labeled carrier proteome has been developed to boost MS signal and increase throughput (9, 16, 17). In line with this concept, increasing studies have underscored the importance of signal boosting using a carrier proteome channel, indicating a trade-off must be made between the depth of peptide identification and the quantification accuracy (18, 19, 20). To alleviate this issue, several factors, including the proper cell number of carrier channel, instrument settings, isotope impurities, and the feasibility of SNR and intensity in data interpretation, have been therefore system-wise addressed (20). Based on these improvements, several protocols were established for enhancing the quantitative accuracy and consistency, such as SCoPE2 (21) by importing automated and miniaturized sample preparation and pSCoPE (13, 22) by introducing the analysis of prioritized peptides.

Alternatively, the retention time alignment of ions with the same MS1 features is well-appreciated for robust protein identification and quantification in proteomics. Several such approaches have been so far invented, such as MaxQuant/IonQuant match-between-runs (MBR) (23, 24), Skyline ion matching (25), and DeMix-Q (26), to transfer peptide identifications and to increase the quantifiable data points by integrations of MS1 features across samples. These algorithms have long been utilized to overcome the semi-stochasticity of data-dependent acquisition analysis that not all peptides are chosen for fragmentation consistently across the replicate experiments. In 2019, the DART-ID (Data-driven Alignment of Retention Times for IDentification) method, using Bayesian frameworks for retention time alignment, demonstrated the improvement in the SCP proteome coverage by elevating the confidence in peptide-spectrum-matches (PSMs) (27). In line with this concept, the MaxQuant MBR is further strengthened enabling reducing the missing value across isobaric labeling batches, called isobaric MBR (IMBR), as a multiplexed approach is crucial for MS signal amplification, especially for trace samples (28). Though stable isobaric labeling with carrier proteome boosting has become an important tool to investigate SCP, yet, the information on IMBR effect across multiplexed datasets and batch effect normalization for identifying meaningful proteins is limited. To take advantage of signal boosting using isobaric labeling, the issues of missing value across multiplexed datasets and batch effect normalization have to be addressed to identify the correct DEPs. In this study, we, therefore, aim to extensively explore the effect of IMBR and the normalization settings underlying the recommended isobaric carrier experimental design in SCP in order to determine accurate biologically significant proteins (20, 29).

## Experimental Procedures

### Experimental Design and Statistical Rationale

To comprehensively evaluate the effects of IMBR, quantification quality control, PSM-level normalization, and imputation tuning, the samples from eAL00219 to eAL00266 in the pSCoPE study (MassIVE: MSV000089159) were downloaded and processed with MaxQuant to form the SC dataset in this study (13). It is composed of 48 TMTpro18-plex batches, including 313 melanoma cells (WM989-A6-G3) and 315 monocytes (U-937). The sample annotation is followed the supplemental Table S1 of https://scp.slavovlab.net/Leduc_et_al_2022 (13). This SCP dataset was used to compare the analysis results with or without applying IMBR and PSM-level normalization. Moreover, to compare the DEPs derived from SCP and bulk-cell proteomics (BCP), two TMTpro 18-plex BCP datasets (wAL00400 and wAL00401) from the identical pSCoPE study with the same cell lines were also downloaded and processed using MaxQuant. The batch effect was relieved by PSM-level weighted ratio normalization or protein-level normalization according to the comparison. DEPs were obtained using the Limma built-in differential expression (DE) analysis with a cutoff *p*-value ≤0.05 (30). The workflow is depicted in supplemental Fig. S1.

### Reagents and Antibodies

Chemical reagents including sodium deoxycholate (SDC), sodium lauroyl sarcosine (SLS), Tris-HCl, sodium dodecyl sulfate (SDS), medium of MCDB 153 and Leibovitz L-15, and Tween-20 were purchased from Sigma Aldrich. Protease inhibitor cocktails were obtained from BIOTOOLS. RPMI1640 culture medium, 100 units/ml penicillin, 100 μg/ml streptomycin, and Trypsin-EDTA were bought from Gibco (Thermo Fisher Scientific, Inc). Fetal bovine serum was purchased from Cytiva. Polyvinylidene difluoride (PVDF, 0.45 μm pore size) membranes and enhanced chemiluminescent substrate was gained from PerkinElmer. The primary antibodies against UCH-L3 (12384-1-AP), CYC1 (10242-1-AP), RAC (24072-1-AP), UFM1 (15883-1-AP) were purchased from ProteinTech, while against S100A10 (A13614) and GNG12 (PA529207) were obtained from Abclonal and Invitrogen, respectively. Secondary anti-rabbit HRP-conjugated antibody (RA-BZ202) were obtained from Croyez Bioscience.

### Cell Culture

A human pro-monocytic myeloid leukemia cell line U937 was obtained from the American Type Culture Collection (ATCC) and the melanoma cells (WM989) were purchased from ROCKLAND. U937 cells were maintained in suspension culture in RPMI-1640 supplemented with 10% heat-inactivated fetal bovine serum and 1% penicillin-streptomycin. The cells were grown at 37 °C in 5% CO2 in a humidified atmosphere and sub-cultured every third or fourth day. WM989 cells were grown as adherent cultures in TU2% media which is composed of 80% MCDB 153, 10% Leibovitz L-15, 2% fetal bovine serum, and 0.5% penicillin-streptomycin, and 1.68 mM calcium chloride. Cells were passaged at 80% confluence using 0.25% Trypsin-EDTA and re-plated at 30% confluence.

### Cell Sorting

Cells were washed with normal phosphate saline and resuspended in phosphate saline containing 2 mM EDTA and 2% FBS at the cell density of 3 × 10^∧^7 cells/ml. After staining with propidium iodide solution, we utilized restricted gating cell sorting (BD, FACSAria III) with FSC/SSC and PI (propidium iodide)-negative parameters to exclude cell debris, doublets, aggregates, and unsuitable cells. For immunoblotting analysis, exact 2 × 10^∧^5 cells were pooled as one sample for cell lysis and protein extraction.

### Data Processing

All the downloaded MS raw files were processed using MaxQuant (ver.2.2.0.0) with its built-in search engine, Andromeda, for peptide/protein identification and quantification (31, 32). Tandem mass spectra were searched against the SwissProt *Homo sapiens* database (26,011 protein sequence entries, downloaded on May 19, 2020) and common contaminants for protein identification and quantification (23, 32). The default MS2 reporter ion quantification parameters with TMTpro 18-plex label as fixed modifications on lysine and peptide N-termini were utilized with minor adjustments (23). In brief, the initial mass tolerance was 20 ppm for precursor ions and 20 ppm for fragment ions with trypsin specificity allowing up to two missed cleavages. The oxidation of methionine and protein N-terminal acetylation were set as variable modifications and cysteine carbamidomethylation was set as fixed modification. PSM and protein false discovery rates were both applied at 1%. MBR function was set to on or off depending on the comparison conditions described in the main text. PSM-level weighted ratio normalization was included for comparing the performance of batch effect removal (28). The MaxQuant protein and peptide quantification tables are available at https://github.com/Sung-Huan/SCP_quantification_data.

### Data Analysis

The workflow is illustrated in supplemental Fig. S1. In brief, all the steps were completed using Perseus software (ver. 2.0.10.0) (33). The proteins labelled as reverse, potential contaminant or only identified by site were removed for excluding the unreliable proteins. Logarithm at Log 2 scale was performed on the data for further analysis. To group the SCP and BCP samples based on the study of Leduc *et al.*, two annotation tables (available at https://github.com/Sung-Huan/SCP_quantification_data) were generated for performing group annotations on Perseus. The channels of reference, control, carrier and unused are removed for the further analyses. The criteria of missing value filtering in SCP dataset at protein level or sample level was optimized by griding analysis, while in BCP dataset, the proteins containing ≥30% missing values, as a conventional threshold, across all channels were excluded. In our previous work, the Perseus default imputation has demonstrated its outperformance on other imputation algorithms (34). Here, we therefore optimized the down-shift parameters of Perseus imputation for adapting the SCP dataset. Furthermore, several normalization approaches, including PSM-level ratio weighting and protein-level ComBat or Limma were applied to remove batch effect, respectively. PSM-level normalization contains several steps to generate protein quantifications. The procedures and an example of normalization can be seen in the follow equations and supplemental Fig. S2.

(1) n=thetotalnumberofPSMsfromaprotein

(2) $$X=\left[list\;of\;PSM\;Intensity\right]=\left[x_{1\;},x_{2\;},x_{3\;}\ldots x_{n\;}\right]$$

(3) $$R=\left[Intensities\;of\;reference\;channels\right]=\left[r_{1\;},r_{2\;},r_{3\;}\ldots r_{n\;}\right]$$

(4) $$\left\{ \begin{array}{c} if\;reference\;channels\;existed,{Xr}_{i}=\frac{X_{i}}{R_{i}}\\ without\;reference\;channels,{Xr}_{i}=\frac{X_{i}}{\sum_{j=1}^{n}X_{j}} \end{array}\right.,for\;i=1,2,3,\ldots ,n$$

Equations (1), (2), (3) defines n, X and R indicate the total number of PSMs from a protein, their intensities and the corresponding reference channel intensities, respectively. The Xr represents the ratio of PSM intensities which are divided by the intensity of their corresponding reference channels from the TMT-multiplexing set. If the dataset contains no reference channel, the sum of intensities from all channels is used for the normalization (Equation 4). Moreover, the multiplies of precursor ion intensities and MS/MS fill time are utilized as weighting factors. This is intended to be proportional to the quantity of ions subjected to fragmentation. The multiplied values are further exponentiated with 0.75, which is optimized by Yu *et al.* (28) 2020 (Equation 5). Afterward, the weighting factors are normalized based on the sum of all its elements (Equation 6).

(5) $$F_{i}={\left(Precursor\;intensity\times MSMS\;fill\;time\right)}^{0.75},for\;i=1,2,3,\ldots ,n$$

(6) $${Fr}_{i}=\frac{F_{i}}{\sum_{j=1}^{n}F_{j}},for\;i=1,2,3,\ldots ,n$$

$F_{i}$ and ${Fr}_{i}$ means the raw and the normalized weighting factors for the i spectrum, respectively. Following this, the list of Xr is sorted in ascending order (Equation 7). The normalized weighting factors (Fr) are also arranged according to the same order as the sorted Xr (Equation 8).

(7) Xs=Sort(Xr)

(8) W=Arrangment(Fr,SortOrder(Xr))

Here, Xs indicates the list of the sorted normalized PSM intensities (Xr) in ascending order. The function SortOrder(Xr) returns the order of PSM intensities after sorting Xr. Using this order, the normalized weighting factors (Fr) are arranged accordingly, and the resulting arrangement Arrangment(Fr,SortOrder(Xr)) is denoted as W. To generate protein intensity, weighted median was applied. Weighted median is the k th element of W which k needs to meet the condition as Equation 9.

(9) (∑i=1k−1wi≤12)∩(∑i=k+1n≤12)

The protein intensities are then assigned as the k th normalized PSM intensity of Xs (Equation 10).

(10) $$Protein\;intensity={Xs}_{k}$$

Furthermore, for comparing normalization performance, the built-in ComBat or Limma algorithms in Perseus were also applied to the SCP dataset without PSM-level weighted ratio normalization (supplemental Fig. S3). After imputation, Limma DE analysis was employed to identify DEPs from both SCP and BCP datasets.

### Immunoblotting Analysis

Cells were lysed with lysis buffer containing 12 mM SDC, 12 mM SLS, 100 mM Tris-HCl (pH9.0), and a complete EDTA-free protease inhibitor cocktail. Proteins from equal cell numbers were loaded into 15% SDS-polyacrylamide gel followed by transferring to PVDF membranes. Membranes were blocked with 5% BSA in TBS containing 0.1% Tween-20 (TBST), probed with indicated primary antibodies at the ratio of 1/1000 (v/v), detected with HRP-conjugated secondary antibodies (1/3000, v/v), visualized by enhanced chemiluminescent substrate, and imaged using ChemiDoc Imaging System (Bio-Rad Laboratories). The quantitative densitometry of immunoblotting was performed by ImageJ 1.53 t (National Institutes of Health).

## Results

Compared to BCP, the stochastic effect of the data-dependent acquisition mode compromises the quantification capability more severely in SCP analysis. The presence of significantly more missing values and higher signal variation pose challenges for data analysis in terms of identifying biologically meaningful proteins, especially those that are relatively low-expressed. While the signal boosting provided by multiplexed isobaric labeling has been widely acknowledged, the applicability of the current data processing and analysis workflow for SCP has not been comprehensively assessed, primarily because most of these methods are developed based on BCP principles (28). We aimed to figure out the optimal way to analyze the SCP dataset by reevaluating the impact of IMBR, quantification filtering, and data normalization. To achieve this, we utilized the published dataset from the pSCoPE work (13), which comprised 628 SCP analyses from two distinct cell types: 313 melanoma cells and 315 monocytes, using the TMTpro18-plex approach. All MS raw files underwent processing with the public MaxQuant software (23, 32), both with or without IMBR function and PSM-level normalization for protein identification and quantification (supplemental Fig. S1). Proteins identified based on a single PSM were also considered for peptide/protein identification; however, all of them were excluded from DEP analysis due to the presence of substantial missing values. The annotated spectra of these cases were provided to support the identification confidence (supplemental Fig. S4). Simultaneously, the BCP datasets from the identical work were also included to compare the efficiency of DEP identification.

### IMBR Relieved the Missing Value Effect on SCP Analysis

**Fig. 1:**
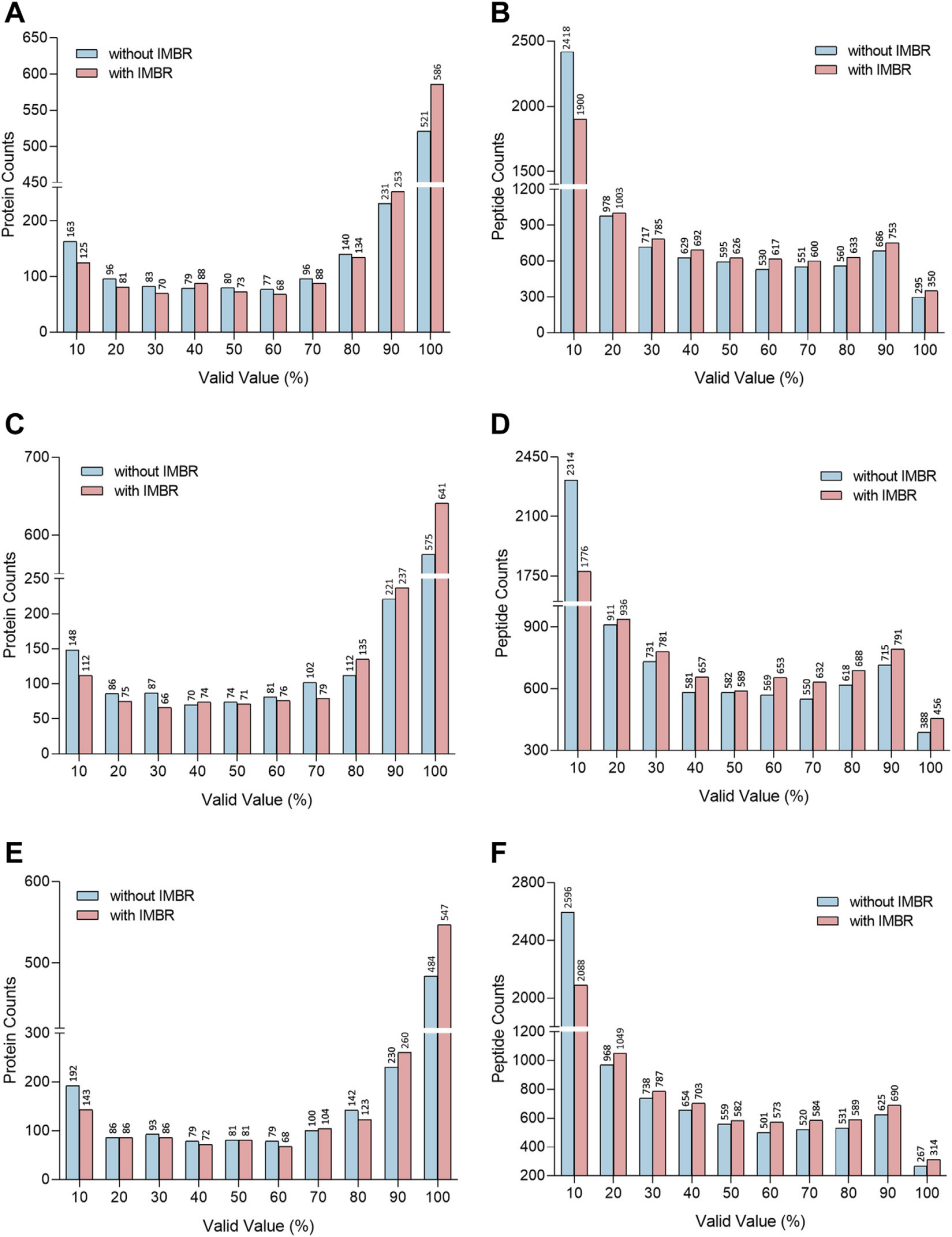
**Comparison of the number of quantified proteins and peptides with or without IMBR assistance.***Pink* and *blue bars* represent the number of quantified (*A*) proteins and (*B*) peptides with or without applying IMBR, respectively, across 48 TMT18plex sets. IMBR significantly improved the number of quantified proteins and peptides in (*C* and *D*) melanoma cells and (*E* and *F*) monocytes.

The persistent issue of missing value in the proteomics field, especially across multiplexed sets, prompts the initial assessment of the IMBR effect in SCP using the public pSCoPE dataset (13). Apparently, when IMBR was applied, the number of evidence entries corresponding to 3D MS1 features with at least one MS/MS spectrum increased significantly from 197,176 to 314,621, representing a 59% enhancement. In contrast, only a 14% increase was observed in the BCP dataset, going from 7334 to 8525. The evidence tables are available at the jPOST repository (https://repository.jpostdb.org) with accession number JPST002464. Furthermore, the implementation of IMBR led to a 12% increase in quantified proteins (from 521 to 586) and a 19% increase in peptides (from 295 to 350) with less than 10% missing values across 48 TMTpro 18-plex batches (Fig. 1, *A* and *B*). On the other hand, poorly quantified proteins and peptides, characterized by over 90% missing values across 48 TMT sets, decreased from 163 to 125 at the protein level (−23%) and from 2418 to 1900 at the peptide level (−21%, Fig. 1, *A* and *B*). As expected, a similar improvement was also found in peptide and protein quantification for individual melanoma and monocyte cell populations (Fig. 1, *C*–*F*). Similar enhancement was also observed in another SCP dataset, which constituted 320 macrophages and 128 monocytes, from the SCoPE2 study (selected samples: 190222S_LCA9_X_FP94AA to FP94BP and 190321S_LCA10_X_FP97AB to FP97BP) (21) (supplemental Fig. S5). Implementing IMBR led to a 16% and 24% increase in the number of quantified peptides and proteins with less than 10% missing values. The count of proteins and peptides containing more than 90% missing values decreased by 14% and 16%, respectively. Reasonably, the rescued quantification values from the matched spectra were mainly due to the missing or low intensity of b/y ions (supplemental Fig. S6). Through the matching of the precursor features, reporter ions, and relatively high b/y ions to the identified PSMs, the peptide intensity from the unidentified spectrum could be therefore quantified. Additionally, the distribution of quantified protein medians, with or without IMBR, showed a similar pattern, indicating that IMBR did not alter the data profile even in the low peptide counts, but significantly improved protein quantification (supplemental Fig. S7). Clearly, the utilization of the IMBR function obviously expanded quantification coverage, underscoring its crucial role in recovering missing values. This enhancement facilitates the identification of biologically important proteins with relatively low abundance during DE analysis.

### Controlling Quantification Quality Enhanced Sample Clustering and the Identification of DEPs

In BCP analysis, common strategies for addressing the limitations of stochastic MS-based proteomics involve removing proteins with high proportional missing values followed by restoring missing values using imputation or MBR. Instead of injecting equal peptide amounts into LC-MS/MS analysis, SCP quantifies the peptides from one individual cell, making cell selection and sample preparation critical for generating informative datasets. Given the current instrument sensitivity and the behind experimental rationale, SCP datasets are even prone to have high proportional missing values in a subset of cells (supplemental Fig. S8), this phenomenon is strikingly different from BCP datasets. A high level of missing values has been widely evident to compromise the statistical power and DEP identification, to solve this issue, removing the proteins with a large number of missing values holds a standardized step in BCP data processing. In general, approximately 80% of identified proteins were retained when applying the conventional criteria of excluding proteins with more than 30% missing values in BCP analysis (supplemental Fig. S9). In SCP datasets, however, only 62% of identified proteins remained, this largely decreased the size of the protein pool available for identifying meaningful DEPs. The reason behind this was not only the randomized ion-acquiring issue but also the presence of low-quality cells containing extensive missing values. These challenges together make the conventional filtering insufficient to eliminate the statistical bias against the quantification results of low-quality cells. We therefore introduced an additional quantification control based on cell quality to mitigate the impact of missing values on statistical analysis.

**Fig. 2:**
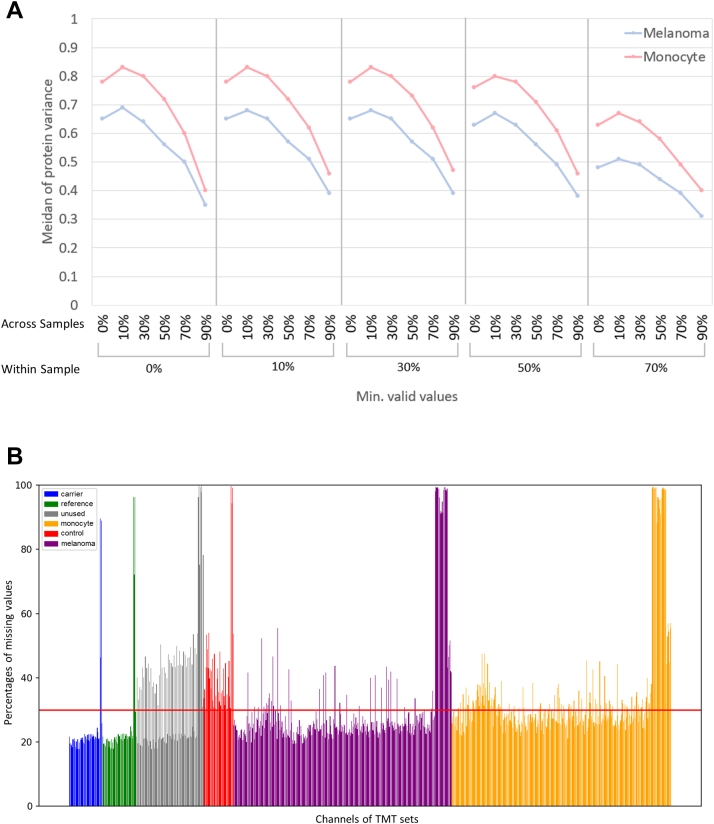
**Assessment of various criteria for quantification quality.***A*, the distribution of median protein variances with different cutoffs of the percentage of valid values within and across samples. The *blue line* represents the median variances calculated from the proteins quantified in melanoma, while the *red line* is from monocytes. *B*, the content of missing values for each TMT channel. Each bar represents the percentage of missing values presented in each sample. Colors mean different sample types, *blue*, carrier; *green*, reference; *red*, control; *yellow*, monocyte; *purple*, melanoma; and *grey*, empty. The control sample means the channels containing all reagents but a cell. The *red horizontal line* represents the defined cutoff (30% of missing value) for the data filtering in this study.

In the quest for optimal filtering, a griding evaluation of cutoffs for missing values was conducted. The results unveiled a substantial decrease in the median of protein variance when the minimum valid value within cells was set as 70%, irrespective of whether conventional filtering regarding the percentage of proteins containing a valid value was applied or not (Fig. 2*A*). As anticipated, a more stringent threshold for the minimum valid value across cells resulted in greater stability in protein variance (Fig. 2*A*). Attempting to increase the threshold for the minimum valid value within cells to 90%, unfortunately, yielded no retained proteins. Notably, with the necessity of at least 70% of valid values within a sample, all control channels without any cells were removed, leaving 434 cells representing around 70% of the sample size (Table 1 and Fig. 2*B*). These results indicated that setting the criterion at 70% of the minimum valid value within cells was appropriate for fine-tuning the subsequent filtering criteria. With this threshold, a significant reduction in protein counts occurred when the minimum valid values at protein level were set larger than 50%, resulting in the removal of more than 30% of identified proteins, including some constantly expressed proteins in melanomas/monocytes, such as GAPDHS (Glyceraldehyde-3-phosphate dehydrogenase) in melanoma cells (35) and DUSP3 (Dual specificity protein phosphatase 3) in monocytes (36), respectively (Table 1 and supplemental Table S1). Therefore, the threshold of 50% of minimum valid value across samples exhibited as a suitable criterion that up to 76% of proteins were kept, which is similar to the BCP dataset (Table 1). Taken together, these results established the minimum valid value within a sample (cell) and across samples (protein) as 70% and 50%, respectively.

**Table 1:** The number of remaining proteins and cells with different quantification quality filtering with IMBR function

Minin. Valid values	Protein counts						Cell counts
Proteins	0%	10%	30%	50%	70%	90%	
Cells							
70%	1566	1458	1328	**1197**	1064	839	434
50%	1566	1448	1306	1161	1017	767	580
30%	1566	1446	1303	1156	1011	745	589
10%	1566	1446	1303	1156	1011	745	589
0%	1566	1437	1289	1127	973	581	628

Bold value indicates the optimal filtering setting in this study.

**Fig. 3:**
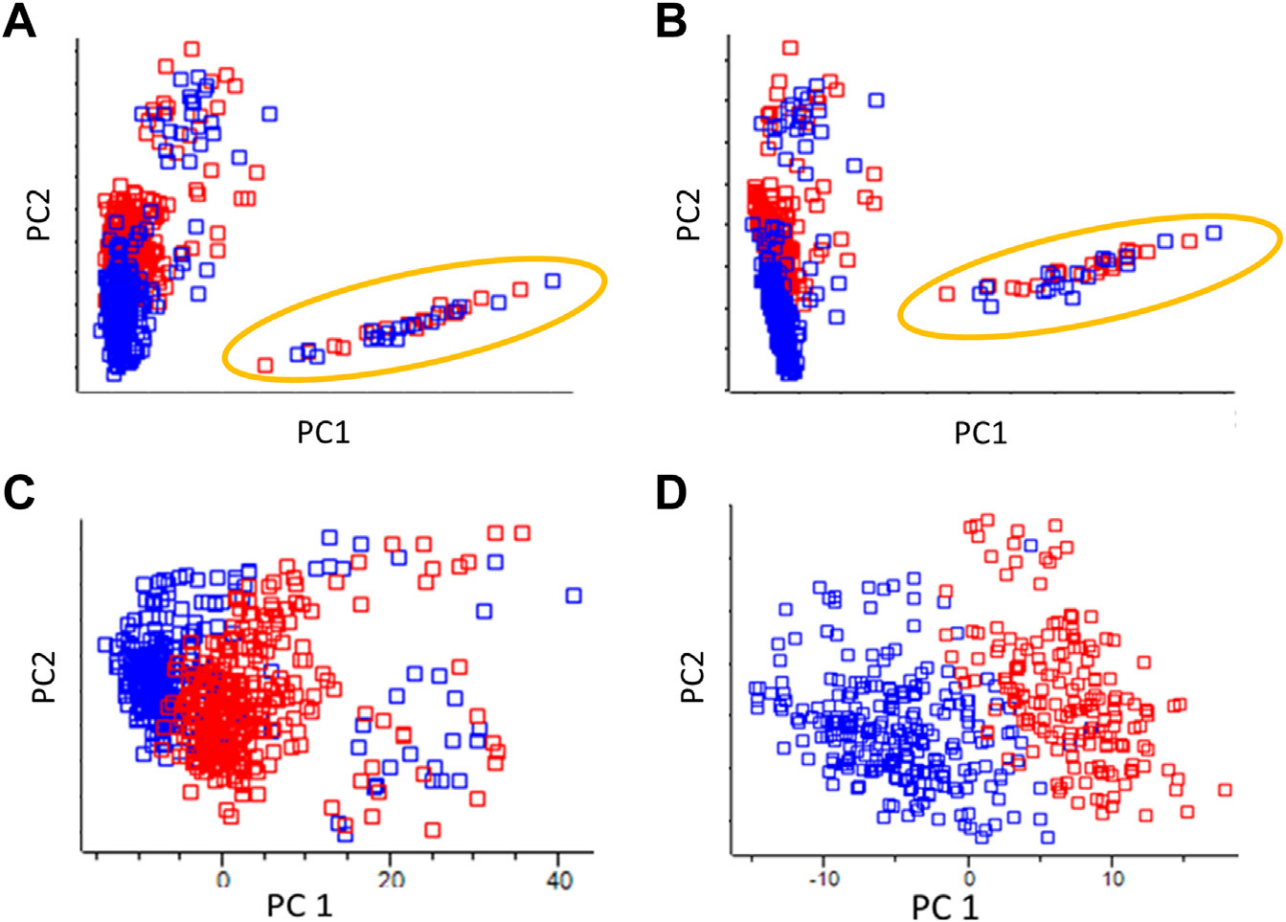
**Principal component analysis with different quantification quality thresholds.** Four different criteria of missing value cutoff were applied: (*A*) 0/0, (*B*) 30/30, (*C*) 0/70, and (*D*) 70/50, where the numbers before and after the slash represent the minimum valid values in a cell and a protein, respectively. *Blue squares* represent the melanoma cells while *red* ones are monocytes. The samples inside the ellipse on (*A*) and (*B*) are from the samples—eAL00263, eAL00264 and eAL00265.

As a proof of concept, we further evaluated the performance of different quantification control approaches: conventional (0/70, percentage of valid values within/across samples), loose (30/30), rigorous (70/50), and without quantification control (0/0). As expected, the rigorous data filtering revealed the most accurate sample clustering in principal component analysis (PCA) and the lowest protein variances (Fig. 3 and supplemental Fig. S10). Notably, these two distinct cell types were unable to be differentiated once the cell quantification filtering was not applied (0/0 and 0/70 in Fig. 3). Furthermore, the data distributions of three TMTpro 18-plex sets (eAL00263 - eAL00265) with extremely low quantification quality were significantly influenced by the methods of data filtering. This impact was alleviated when cell quality was controlled (30/30 and 70/50, supplemental Fig. S11). Although loose filtering (30/30) has already shown improvement in cell type separation, rigorous filtering (70/50) achieved both more evident cell type segregation and reduced protein variance (Fig. 3 and supplemental Fig. S10). Consequently, controlling quantification quality within and across cells emerged as a critical step for performing accurate DE analysis, resulting in a noticeable improvement in SCP analysis. Furthermore, as a synergistic approach, the combined usage of IMBR for missing value restoration, along with rigorous quantification control, has proven essential for elevating the overall robustness and reliability of SCP analysis (Tables 1 and 2).

**Table 2:** The number of remaining proteins and cells with different quantification quality filtering without IMBR function

Min. Valid values	Protein counts						Cell counts
Proteins	0%	10%	30%	50%	70%	90%	
Cells							
70%	1566	1441	1293	1166	1013	796	259
50%	1566	1411	1236	1103	949	706	572
30%	1566	1410	1235	1097	929	675	589
10%	1566	1410	1235	1097	929	675	589
0%	1566	1404	1224	1065	892	521	628

### Adequate Quantification Control Alleviated the Need for Imputation Adjustment

**Fig. 4:**
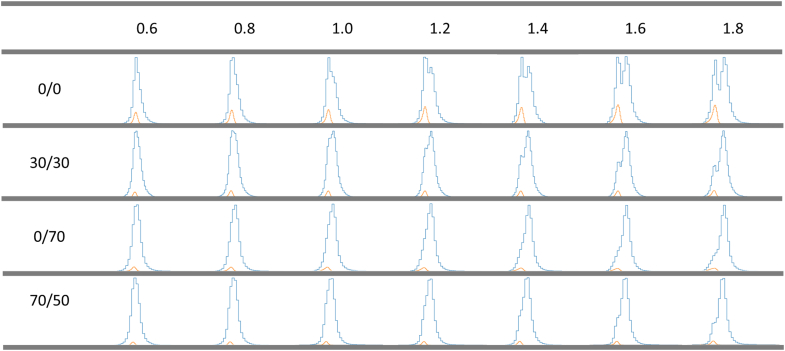
**Histogram of SCP dataset using varied imputation down-shift with different quantification quality control cutoffs.** The first row indicates the down-shift values applied in imputation. From the second to fifth row are the data distribution after imputation under four different cutoffs of missing values – 0/0, 30/30, 0/70 and 70/50 (the numbers before and after the slash represent the minimum valid values in a cell and a protein, respectively). The *blue* and *orange* histograms represent the distribution of protein intensities from the whole dataset and imputation, respectively.

Left-censored missing data (LCMD) imputation algorithm has been reported as an effective method to deal with the replenishment of the missing values for DEP identification in BCP analysis (34). Amongst, the normal distribution-based imputation by Perseus default settings has shown its outperformance compared with other imputation methods in our previous work (34). Given the significantly higher prevalence of missing values in the SCP dataset, we further assessed the applicability of normal distribution-based imputation in DE analysis. Using the default settings in Perseus, the imputed peaks were distinctly visible, particularly in the dataset lacking adequate quantification control (Fig. 4, and supplemental Fig. S12, *A* and *B*), thereby distorting the normal distribution and thus compromising statistical power. This led us to smoothly adjust the down-shift from 1.8 to 0.6, representing the standard deviation multiplier of the valid data used for the downward shift in order to fit the smooth normal distribution. Obviously, the impact of imputed peaks on the normal distribution decreased as the down-shift value decreased (Fig. 4). Of note, when the down-shift was less or equal to 1.0, the imputed peaks no longer deteriorated the normal distribution, even in the absence of quantification control (Fig. 4 and supplemental Fig. S12). Although the adjustment of the down-shift value for imputation may not be as crucial for datasets with quantification quality control, moderate tuning, and data distribution inspection are still necessary to maintain statistical power. These results support our abovementioned findings that controlling the quantification quality is indeed a demanding step in enhancing the accuracy of data analysis.

### PSM-Level Normalization Preserved Original Data Profiles With on Par Performance to Protein-Level Normalization

Given the limitation of multiplexing capabilities, capped at a maximum of 18-plex, normalization therefore plays a critical role in batch effect removal. In this context, we included PSM-level weighted ratio normalization, along with commonly used Limma and ComBat protein-level normalizations for comparison (28, 30, 37, 38, 39). As expected, the employment of normalization, whether at the PSM level or protein level, conspicuously improved cell type separation with rigorous filtering (70/50, supplemental Fig. S13). Furthermore, the accuracy of sample grouping was significantly elevated with any of these normalization methods, exhibiting approximately 21% to 23% improvement compared to the dataset without normalization (supplemental Fig. S14). However, we did observe that Limma and ComBat were not as sensitive as PSM-level normalization to data quality, as cell groupings remained almost identical regardless of data filtering (supplemental Fig. S15). This difference can be explained by the inclusion of imputed values for normalization, causing the loss of the original dataset pattern in the protein-level normalization approaches. In contrast, PSM-level normalization preserved the original dataset profiles, enabling us to assess data quality and thereby avoid misleading analysis results. Remarkably, coupling proper quantification filtering with PSM-level normalization exhibited as an effective way to process SCP data. This combination proved indispensable, mutually complementing each other, and effectively enhancing the accuracy and reliability in data analysis.

### Characterization and Validation of DEPs Identified by Refined Data Analysis Pipeline

**Fig. 5:**
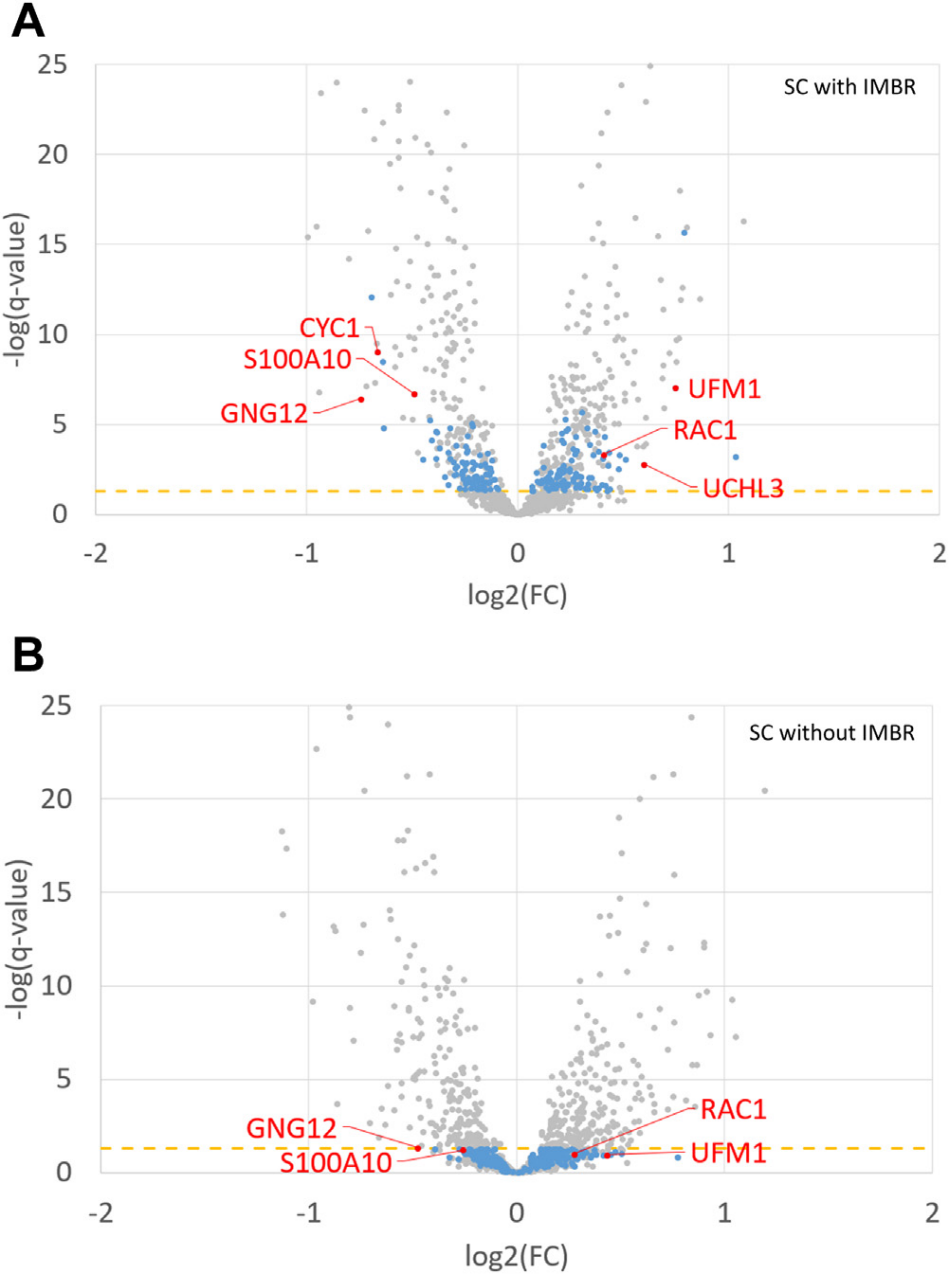
**Volcano plots of the SCP dataset with the refined pipeline.***A*, the SCP dataset was processed with IMBR, quantification quality control, and PSM-level normalization. *B*, the SCP dataset was processed with quantification quality control and PSM-level normalization but IMBR. *Blue dots* represent the DEPs uniquely identified in the dataset with IMBR functionality. *Red dots* are the proteins selected for validation. The *yellow dashed line* indicates the position of the *q*-value is 0.05.

**Fig. 6:**
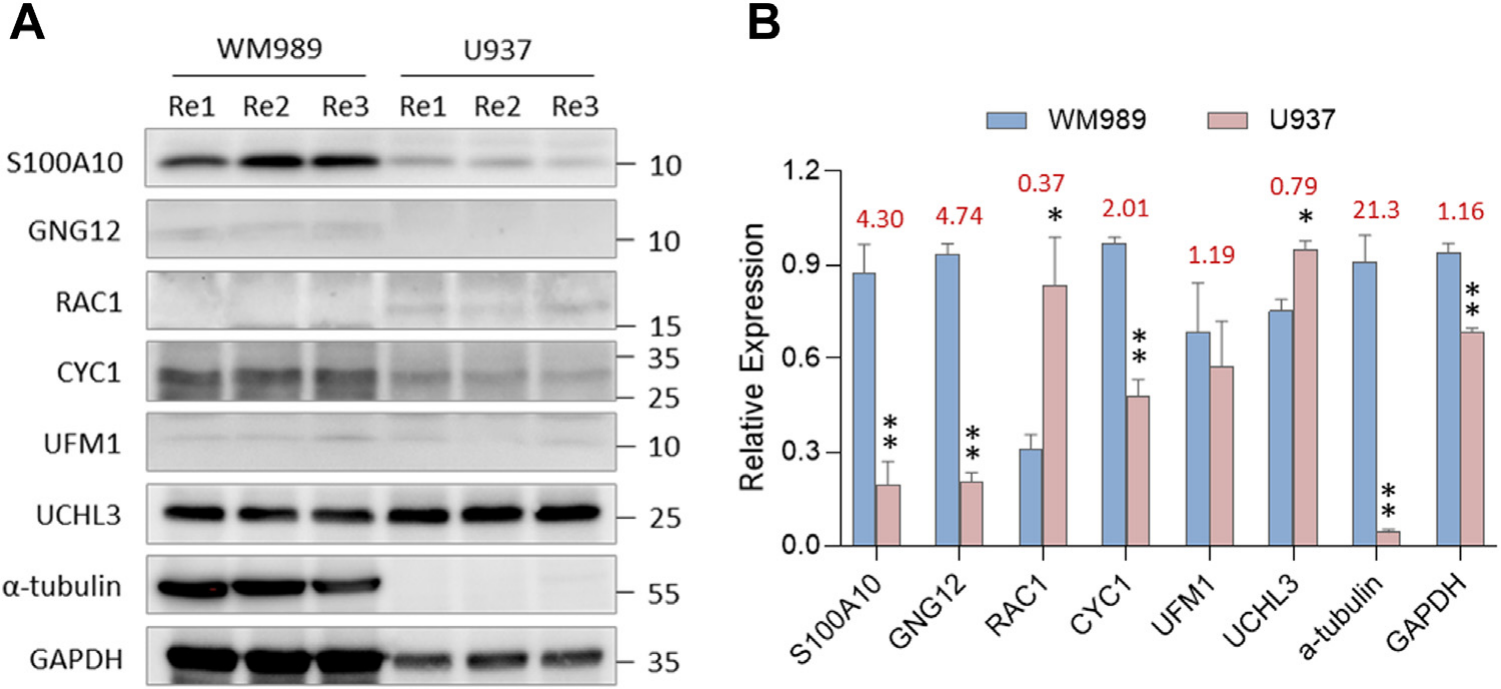
**Immunoblotting analysis of the DEPs identified in SCP dataset.** Equal melanoma and monocyte cell numbers were lysed and protein extracts were loaded for SDS-PAGE analysis followed by western blotting. *A*, relative protein abundances of six selected targets were analyzed using western blotting. *B*, quantification of western blotting results using Image J. Error bars indicate ±S.D.E. Statistical significance is denoted by asterisks, where ∗ and ∗∗ denote *p* ≤ 0.05 and *p* ≤ 0.01, respectively. Values in red are the protein expression level relative to that expressed in U937 cells.

DE analysis is one of the most commonly used methods for discovering the key factors between sample groups. SCP proves advantageous in identifying DEPs specific to individual cell populations. A large overlap of identified proteins between SCP and BCP datasets was foreseen and the higher number of identified proteins in SCP was attributed to multiple sample batches (supplemental Fig. S16*A*). In total, 666 out of 1566 quantified proteins were significantly altered between these 2 cell types and most of the DEPs, approximately up to 51%, were co-identified in the parallel BCP analysis (supplemental Fig. S16*B*). As illustrated by DEPs uniquely identified in SCP with IMBR, the q-values were apparently decreased due to the effective missing value restoration by IMBR, thereby enhancing DEP identification (Fig. 5). In order to validate the DEPs identified in the SCP dataset, six DEPs detected from the SC IMBR dataset with low q-values were selected for immunoblotting analysis (supplemental Table S2). Among these six DEPs, five targeted proteins showed a consistent trend with the MS-based SCP result (Fig. 6). Interestingly, only two proteins exhibited difference when equal protein inputs were loaded, and the differences were not as apparent as that observed in immunoblotting results with equal cell numbers (supplemental Fig. S17). These results indicated that the inherent variance of individual cells could be average by mixing them as BCP analysis. Together with the precursor interference effect (40), these factors collectively impede the detection of DEPs in MS-based BCP analysis. S100A10 and GNG12 proteins are known to play roles in melanoma malignancy (41, 42, 43) and the expression levels of these two proteins were higher in WM989 melanoma cells from both BCP and SCP datasets. In contrast, CYC1 was exclusively overrepresented in the SCP dataset with IMBR, which is a component of complex III of the electron transport chain in mitochondrial membrane. Perturbating this electron shuttling causes oxidative stress accumulation accelerating melanin synthesis and melanoma formation (44, 45). On the other hand, the higher expression level of RAC1 and UCHL3 proteins implied the importance of Rho GTPases activity and ubiquitin system in initiating phagocytosis as the first defense line (46, 47). The inconsistency of UFM1 protein between SCP and immunoblotting results may be explained by the quality of available antibody or presence of post-translational modifications. Taking above together, coupling the IMBR function and PSM-level normalization obviously reduced the impairment of missing value, retained data pattern, simplified the data processing steps, and improved the DEP identification, this can benefit the data analyses of SCP.

## Discussion

The significant increase in protein counts without missing values indicated the superior performance of IMBR in protein and peptide quantifications, laying the groundwork for advancing the DEP identification in SCP analysis (Fig. 1). Aligned with previous observations, IMBR increased the number of proteins and peptides with less than 10% missing values by 12% and 19% across 48 TMTpro-18plex batches, respectively. Moreover, when categorized by cell types, proteins and peptides with ≥90% valid values increased by 11% and 18% in melanomas, and 13% and 18% in monocyte cells. As a result of effective missing value restoration, the number of quantified proteins was augmented, with the majority of protein categories being jointly determined within the same cell types, thus expanding the pool of eligible proteins for subsequent DE analysis. Interestingly, the distribution of protein abundances for DEPs uniquely identified in the IMBR SCP dataset was similar to all identified proteins and DEPs in BCP (supplemental Fig. S18). This suggests that the IMBR functionality is essential not only for rescuing quantification values of low-abundance DEPs but also for certain DEPs with high reporter ion intensity. In other words, IMBR plays a crucial role in accurately quantifying proteins, regardless of their abundance levels. On the other hand, due to the limited proteome coverage in SCP analysis, missing value filtering criteria are usually lenient or sometimes not applied at all. However, we made a crucial discovery that the quality control at both cell and protein levels is of important for improving quantification accuracy. Here, the minimum valid values for protein and cell filtering were set at 50% and 70%, respectively, retaining 70% and 76% of cells and identified proteins. These cutoff values were tailored based on the depth and coverage of the input dataset, the background information provided by control channels, and the behavior of BCP datasets. The application of rigorous filtering (70/50) led to significant improvements in sample clustering and protein variance (Fig. 3 and supplemental Fig. S10). Nevertheless, it is important to note that the quantification quality control criteria should be fine-tuned for each specific dataset, as they are highly dependent on the dataset’s unique characteristics. Fine-tuning these values becomes imperative to accurately determine DEPs for SCP analysis.

Currently, the Limma or ComBat algorithm is widely applied to normalize batch effects at the protein level across multiple LC-MS/MS analyses in both BCP and SCP fields. In comparison, the PSM-level weighted ratio normalization showed no significant difference between Limma and ComBat once the appropriate data quality control and imputation were applied (supplemental Figs. S13 and S14). Yet, it should be noted that protein-level normalization using Limma or ComBat includes imputed values, posing challenges in evaluating data quality. This was evident from the fact that no difference was observed with varying missing value cutoffs (supplemental Fig. S15). On the contrary, PSM-level normalization preserved the data distribution closer to the actual dataset, making it more suitable for SCP analysis. In summary, while Limma and ComBat are commonly used, PSM-level normalization emerges as a promising alternative for SCP due to its ability to better retain the real dataset characteristics and enable a more accurate evaluation of data quality.

Collectively, this study presented a significant advancement in SCP data analysis through the combination of IMBR, quantification quality control, imputation adjustment, and PSM-level weighted ratio normalization. Notably, we introduced the concept of quality control, for the first time, demonstrating its effectiveness in diminishing quantification variation, enhancing statistical power, and decreasing reliance on imputation adjustments. The impact of IMBR and PSM-level normalization was further evident in DE analysis, where IMBR unique DEPs were highly relevant to melanoma regulation. In conclusion, these integrated approaches provided significant benefits for multiplexed SCP studies. By adhering to the workflow established in this study, a standardized and efficient pipeline for SCP data analysis can be implemented.

## Data Availability

All the pSCoPE raw data and search results we used for evaluating the SCP analysis pipeline in this study have been deposited in the jPOST repository (https://repository.jpostdb.org) with the accession number of JPST002464 for jPOST and PXD048595 for ProteomeXchange (13, 48). The samples from eAL00219 to eAL00266 were selected to form the SC dataset, and wAL00400 and wAL00401 were utilized to comprise the BCP dataset. The quantification table protein quantification tables for the analyses conducted by IMBR with PSM-level normalization, IMBR solely, control and BCP can be found in supplemental Tables S3–S6. The quantification tables from MaxQuant are also available at https://github.com/Sung-Huan/SCP_quantification_data.

## Supplemental Data

This article contains supplemental data.

## Conflict of interest

The authors declare that they have no conflicts of interest with the contents of this article.

## References

[1] Huang S. (2009). Non-genetic heterogeneity of cells in development: more than just noise. Development.

[2] Weber M., Gerber H. (1991). [Acute cervical syndrome in chondrocalcinosis. 3 elderly patients with calcifications of the transverse atlantis ligament]. Schweiz. Med. Wochenschr..

[3] Liu Y., Beyer A., Aebersold R. (2016). On the dependency of cellular protein levels on mRNA abundance. Cell.

[4] Mahdessian D., Cesnik A.J., Gnann C., Danielsson F., Stenstrom L., Arif M. (2021). Spatiotemporal dissection of the cell cycle with single-cell proteogenomics. Nature.

[5] Vogel C., Marcotte E.M. (2012). Insights into the regulation of protein abundance from proteomic and transcriptomic analyses. Nat. Rev. Genet..

[6] Walsh C.T., Garneau-Tsodikova S., Gatto G.J. (2005). Protein posttranslational modifications: the chemistry of proteome diversifications. Angew. Chem. Int. Ed. Engl..

[7] Labib M., Kelley S.O. (2020). Single-cell analysis targeting the proteome. Nat. Rev. Chem..

[8] Genshaft A.S., Li S., Gallant C.J., Darmanis S., Prakadan S.M., Ziegler C.G. (2016). Multiplexed, targeted profiling of single-cell proteomes and transcriptomes in a single reaction. Genome Biol..

[9] Tsai C.F., Zhao R., Williams S.M., Moore R.J., Schultz K., Chrisler W.B. (2020). An improved boosting to amplify signal with isobaric labeling (iBASIL) strategy for precise quantitative single-cell proteomics. Mol. Cell. Proteomics.

[10] (2023). Single-cell proteomics: challenges and prospects. Nat. Methods.

[11] Ctortecka C., Hartlmayr D., Seth A., Mendjan S., Tourniaire G., Mechtler K. (2022). An automated workflow for multiplexed single-cell proteomics sample preparation at unprecedented sensitivity. bioRxiv.

[12] Zhu Y., Piehowski P.D., Zhao R., Chen J., Shen Y., Moore R.J. (2018). Nanodroplet processing platform for deep and quantitative proteome profiling of 10-100 mammalian cells. Nat. Commun..

[13] Leduc A., Huffman R.G., Cantlon J., Khan S., Slavov N. (2022). Exploring functional protein covariation across single cells using nPOP. Genome Biol..

[14] Tsai C.F., Zhang P., Scholten D., Martin K., Wang Y.T., Zhao R. (2021). Surfactant-assisted one-pot sample preparation for label-free single-cell proteomics. Commun. Biol..

[15] Zhu Y., Clair G., Chrisler W.B., Shen Y., Zhao R., Shukla A.K. (2018). Proteomic analysis of single mammalian cells enabled by microfluidic nanodroplet sample preparation and ultrasensitive NanoLC-MS. Angew. Chem. Int. Ed. Engl..

[16] Budnik B., Levy E., Harmange G., Slavov N. (2018). SCoPE-MS: mass spectrometry of single mammalian cells quantifies proteome heterogeneity during cell differentiation. Genome Biol..

[17] Li J., Van Vranken J.G., Pontano Vaites L., Schweppe D.K., Huttlin E.L., Etienne C. (2020). TMTpro reagents: a set of isobaric labeling mass tags enables simultaneous proteome-wide measurements across 16 samples. Nat. Methods.

[18] Cheung T.K., Lee C.Y., Bayer F.P., McCoy A., Kuster B., Rose C.M. (2021). Defining the carrier proteome limit for single-cell proteomics. Nat. Methods.

[19] Specht H., Slavov N. (2021). Optimizing accuracy and depth of protein quantification in experiments using isobaric carriers. J. Proteome Res..

[20] Ye Z., Batth T.S., Ruther P., Olsen J.V. (2022). A deeper look at carrier proteome effects for single-cell proteomics. Commun. Biol..

[21] Specht H., Emmott E., Petelski A.A., Huffman R.G., Perlman D.H., Serra M. (2021). Single-cell proteomic and transcriptomic analysis of macrophage heterogeneity using SCoPE2. Genome Biol..

[22] Huffman R.G., Leduc A., Wichmann C., Di Gioia M., Borriello F., Specht H. (2023). Prioritized mass spectrometry increases the depth, sensitivity and data completeness of single-cell proteomics. Nat. Methods.

[23] Tyanova S., Temu T., Cox J. (2016). The MaxQuant computational platform for mass spectrometry-based shotgun proteomics. Nat. Protoc..

[24] Yu F., Haynes S.E., Nesvizhskii A.I. (2021). IonQuant enables accurate and sensitive label-free quantification with FDR-controlled match-between-runs. Mol. Cell. Proteomics.

[25] MacLean B., Tomazela D.M., Shulman N., Chambers M., Finney G.L., Frewen B. (2010). Skyline: an open source document editor for creating and analyzing targeted proteomics experiments. Bioinformatics.

[26] Zhang B., Kall L., Zubarev R.A. (2016). DeMix-Q: quantification-centered data processing workflow. Mol. Cell. Proteomics.

[27] Chen A.T., Franks A., Slavov N. (2019). DART-ID increases single-cell proteome coverage. PLoS Comput. Biol..

[28] Yu S.H., Kyriakidou P., Cox J. (2020). Isobaric matching between runs and novel PSM-level normalization in MaxQuant strongly improve reporter ion-based quantification. J. Proteome Res..

[29] Gatto L., Aebersold R., Cox J., Demichev V., Derks J., Emmott E. (2023). Initial recommendations for performing, benchmarking and reporting single-cell proteomics experiments. Nat. Methods.

[30] Ritchie M.E., Phipson B., Wu D., Hu Y., Law C.W., Shi W. (2015). Limma powers differential expression analyses for RNA-sequencing and microarray studies. Nucleic Acids Res..

[31] Cox J., Neuhauser N., Michalski A., Scheltema R.A., Olsen J.V., Mann M. (2011). Andromeda: a peptide search engine integrated into the MaxQuant environment. J. Proteome Res..

[32] Cox J., Mann M. (2008). MaxQuant enables high peptide identification rates, individualized p.p.b.-range mass accuracies and proteome-wide protein quantification. Nat. Biotechnol..

[33] Tyanova S., Temu T., Sinitcyn P., Carlson A., Hein M.Y., Geiger T. (2016). The Perseus computational platform for comprehensive analysis of (prote)omics data. Nat. Methods.

[34] Lin M.H., Wu P.S., Wong T.H., Lin I.Y., Lin J., Cox J. (2022). Benchmarking differential expression, imputation and quantification methods for proteomics data. Brief. Bioinform..

[35] Gill J.G., Leef S.N., Ramesh V., Martin-Sandoval M.S., Rao A.D., West L. (2022). A short isoform of spermatogenic enzyme GAPDHS functions as a metabolic switch and limits metastasis in melanoma. Cancer Res..

[36] Singh P., Dejager L., Amand M., Theatre E., Vandereyken M., Zurashvili T. (2015). DUSP3 genetic deletion confers M2-like macrophage-dependent tolerance to septic shock. J. Immunol..

[37] Chen C., Grennan K., Badner J., Zhang D., Gershon E., Jin L. (2011). Removing batch effects in analysis of expression microarray data: an evaluation of six batch adjustment methods. PLoS One.

[38] Johnson W.E., Li C., Rabinovic A. (2007). Adjusting batch effects in microarray expression data using empirical Bayes methods. Biostatistics.

[39] Muller C., Schillert A., Rothemeier C., Tregouet D.A., Proust C., Binder H. (2016). Removing batch effects from longitudinal gene expression - quantile normalization plus ComBat as best approach for microarray transcriptome data. PLoS One.

[40] Ow S.Y., Salim M., Noirel J., Evans C., Wright P.C. (2011). Minimising iTRAQ ratio compression through understanding LC-MS elution dependence and high-resolution HILIC fractionation. Proteomics.

[41] Byrum S.D., Larson S.K., Avaritt N.L., Moreland L.E., Mackintosh S.G., Cheung W.L. (2013). Quantitative proteomics identifies activation of hallmark pathways of cancer in patient melanoma. J. Proteomics Bioinform..

[42] Meghnani V., Wagh A., Indurthi V.S., Koladia M., Vetter S.W., Law B. (2014). The receptor for advanced glycation end products influences the expression of its S100 protein ligands in melanoma tumors. Int. J. Biochem. Cell Biol..

[43] Petersson S., Shubbar E., Enerback L., Enerback C. (2009). Expression patterns of S100 proteins in melanocytes and melanocytic lesions. Melanoma Res..

[44] Kaminski K., Kazimierczak U., Kolenda T. (2022). Oxidative stress in melanogenesis and melanoma development. Contemp. Oncol. (Pozn).

[45] Rosei M.A., Blarzino C., Coccia R., Foppoli C., Mosca L., Cini C. (1998). Production of melanin pigments by cytochrome c/H2O2 system. Int. J. Biochem. Cell Biol..

[46] Li J., Chai Q.Y., Liu C.H. (2016). The ubiquitin system: a critical regulator of innate immunity and pathogen-host interactions. Cell. Mol. Immunol..

[47] Mao Y., Finnemann S.C. (2012). Essential diurnal Rac1 activation during retinal phagocytosis requires alphavbeta5 integrin but not tyrosine kinases focal adhesion kinase or Mer tyrosine kinase. Mol. Biol. Cell.

[48] Okuda S., Watanabe Y., Moriya Y., Kawano S., Yamamoto T., Matsumoto M. (2017). jPOSTrepo: an international standard data repository for proteomes. Nucleic Acids Res..

