# Supplementary material for: Quantification Quality Control Emerges as a Crucial Factor to Enhance Single-Cell Proteomics Data Analysis
Source: Mol Cell Proteomics. 2024 Apr 15;23(5):100768. doi: 10.1016/j.mcpro.2024.100768 (PMC11103571; doi:10.1016/j.mcpro.2024.100768)
Supplement: Supplemental Data [file mmc1.pdf]

Quantification Quality Control Emerges as A Crucial Factor to Enhance Single Cell Proteomics Data Analysis

Sung-Huan Yu[1,2], Shiau-Ching Chen[1], Pei-Shan Wu[3], Pei-I Kuo[3], Ting-An Chen[3], Hsiang-Ying Lee[4,5], Miao-Hsia Lin[3,*]

[1] Institute of Precision Medicine, College of Medicine, National Sun Yat-sen University, Kaohsiung 80424, Taiwan

[2] School of Medicine, College of Medicine, National Sun Yat-sen University, Kaohsiung 80424, Taiwan

[3] Department of Microbiology, National Taiwan University College of Medicine, Taipei 10051, Taiwan.

[4] Department of Urology, Kaohsiung Medical University Hospital, Kaohsiung 80756, Taiwan.

[5] Department of Urology, School of Medicine, College of Medicine, Kaohsiung Medical University, Kaohsiung 80756, Taiwan.

*Correspondence:

Miao-Hsia Lin

Department of Microbiology, College of Medicine, National Taiwan University

miaohsialin1012@ntu.edu.tw

Tel: +886-2-2312-3456 ext.288285

Content

Figures

Fig. S1. Workflow of the data analysis for SCP dataset using IMBR, imputation and PSM-level normalization.

Fig. S2. A step-by-step example of PSM-level normalization to calculate protein abundance.

Fig. S3. The screenshots of the plug-in for Limma and ComBat normalization in Perseus software.

Fig. S4. MS2 spectra of the proteins were identified by single PSM. (Additional attached PDF file.)

Fig. S5. Comparison of protein/peptide quantification with or without IMBR for SCoPE2 dataset.

Fig. S6. Alignment of b/y-ions between identified and IMBR-rescued MS2 spectra.

Fig. S7. Boxplots of normalized protein intensities with or without IMBR.

Fig. S8. Comparison of missing value content between SCP and BCP datasets.

Fig. S9. The protein counts of BCP and SCP dataset with different missing value filtering thresholds.

Fig. S10. The protein variance between different missing value filtering strategies.

Fig. S11. Three TMT batches containing high proportion of missing values.

Fig. S12. The data distribution with and without imputation using the down-shift from 1.0 and 1.8.

Fig. S13. PCA for the dataset with PSM-level, protein-level normalization, or without normalization.

Fig. S14. Comparison of sample clustering using different normalization strategies.

Fig. S15. PCA plots of SCP dataset with Limma, ComBat, or PSM-level normalization under different quantification control conditions.

Fig. S16. Overlap of (A) all identified proteins and (B) differentially expressed proteins (DEPs) between SCP and BCP datasets.

Fig. S17. Immunoblotting analysis of 6 selected DEPs with equal protein inputs.

Fig. S18. Distributions of protein abundance from the DEPs identified in SCP and all identified proteins and DEPs from BCP.

Tables

Table S1. The protein quantification table is highlighted by different missing value cutoffs (Additional attached xlsx file).

Table S2. The *q*-values of the 6 selected proteins for the validation in SCP and BCP dataset.

Table S3. The protein quantification table of SCP dataset using IMBR and PSM normalization (Additional attached xlsx file).

Table S4. The protein quantification table of SCP dataset using IMBR (Additional attached xlsx file).

Table S5. The protein quantification table of SCP dataset without using IMBR and PSM normalization (Additional attached xlsx file).

Table S6. The protein quantification table of BCP dataset (Additional attached xlsx file).

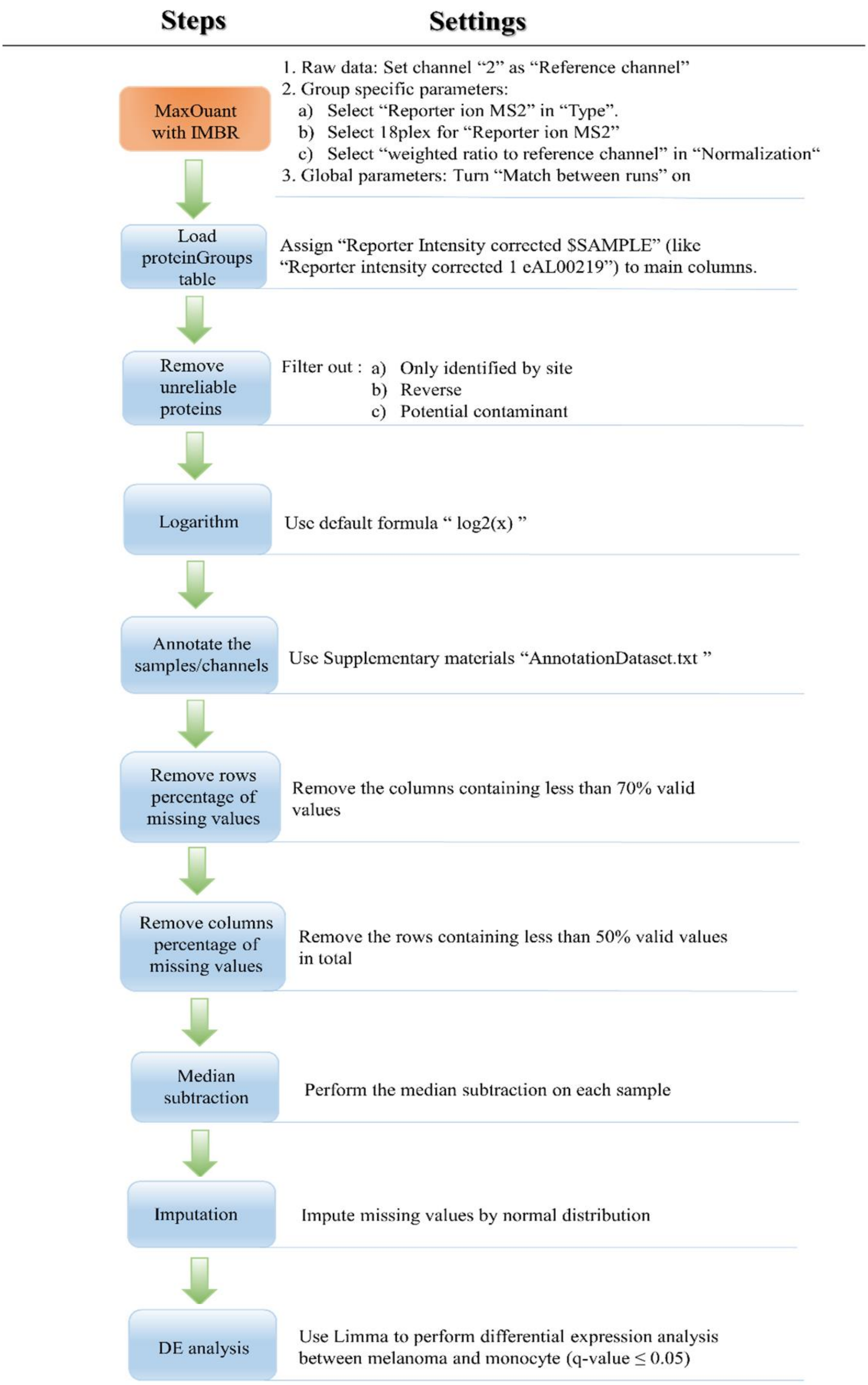

Fig. S1. Workflow of the data analysis for SCP dataset using IMBR, imputation and PSM-level normalization.

PSM	A	B	C	D
	Precursor Intensity	MSMS Fill time	Channel Intensity	Reference Channel Intensity
S1	3800355	30	350.88	2704.15
S2	2081770	56	457.63	2887.56
S3	3532130	31	772.78	4197.28
S4	4530471	36	664.09	3304.18
S5	6439343	44	880.37	4572.88
S6	2219132	50	580.45	3003.51
S7	3018247	47	399.72	2750.56

$(AB)^{0.75}$ C/D

PSM	$(AB)^{0.75}$	C/D
	F = Weighting factor	*Xr* = Ratio
S1	1103339.3	0.130
S2	1121929.5	0.158
S3	1070406.6	0.184
S4	1445375.9	0.201
S5	2183839.3	0.193
S6	1081097.5	0.193
S7	1299838.7	0.145

$$Fr_i = \frac{F_i}{\sum_{j=1}^{n} F_j}, for\ i = 1,2,3, \ldots, n$$

PSM	*Fr*	*Xr*
S1	0.118564349	0.130
S2	0.120562044	0.158
S3	0.115025416	0.184
S4	0.15531945	0.201
S5	0.234674398	0.193
S6	0.116174255	0.193
S7	0.139680087	0.145

Xs = Sort(*Xr*)

PSM	*W*	*Xs*
S1	w_1 = 0.118564349	Xs_1 = 0.130
S7	w_2 = 0.139680087	Xs_2 = 0.145
S2	w_3 = 0.120562044	Xs_3 = 0.158
S3	w_4 = 0.115025416	Xs_4 = 0.184
S5	w_5 = 0.234674398	Xs_5= 0.193
S6	w_6 = 0.116174255	Xs_6= 0.193
S4	w_7 = 0.15531945	Xs_7= 0.201

$\sum_{i=1}^{k-1} w_i \leq 1/2$ and $\sum_{i=k+1}^{7} w_i \leq 1/2$

Weighted median = $w_k = w_4$

PSM	*W*	*Xs*
S1	w_1 = 0.118564349	Xs_1 = 0.130
S7	w_2 = 0.139680087	Xs_2 = 0.145
S2	w_3 = 0.120562044	Xs_3 = 0.158
S3	w_4 = 0.115025416	Xs_4 = 0.184
S5	w_5 = 0.234674398	Xs_5= 0.193
S6	w_6 = 0.116174255	Xs_6= 0.193
S4	w_7 = 0.15531945	Xs_7= 0.201

Assign the ratio to the protein

	Protein intensity
Protein	Xs_4 = 0.184

Fig. S2. A step-by-step example of PSM-level normalization to calculate protein abundance.

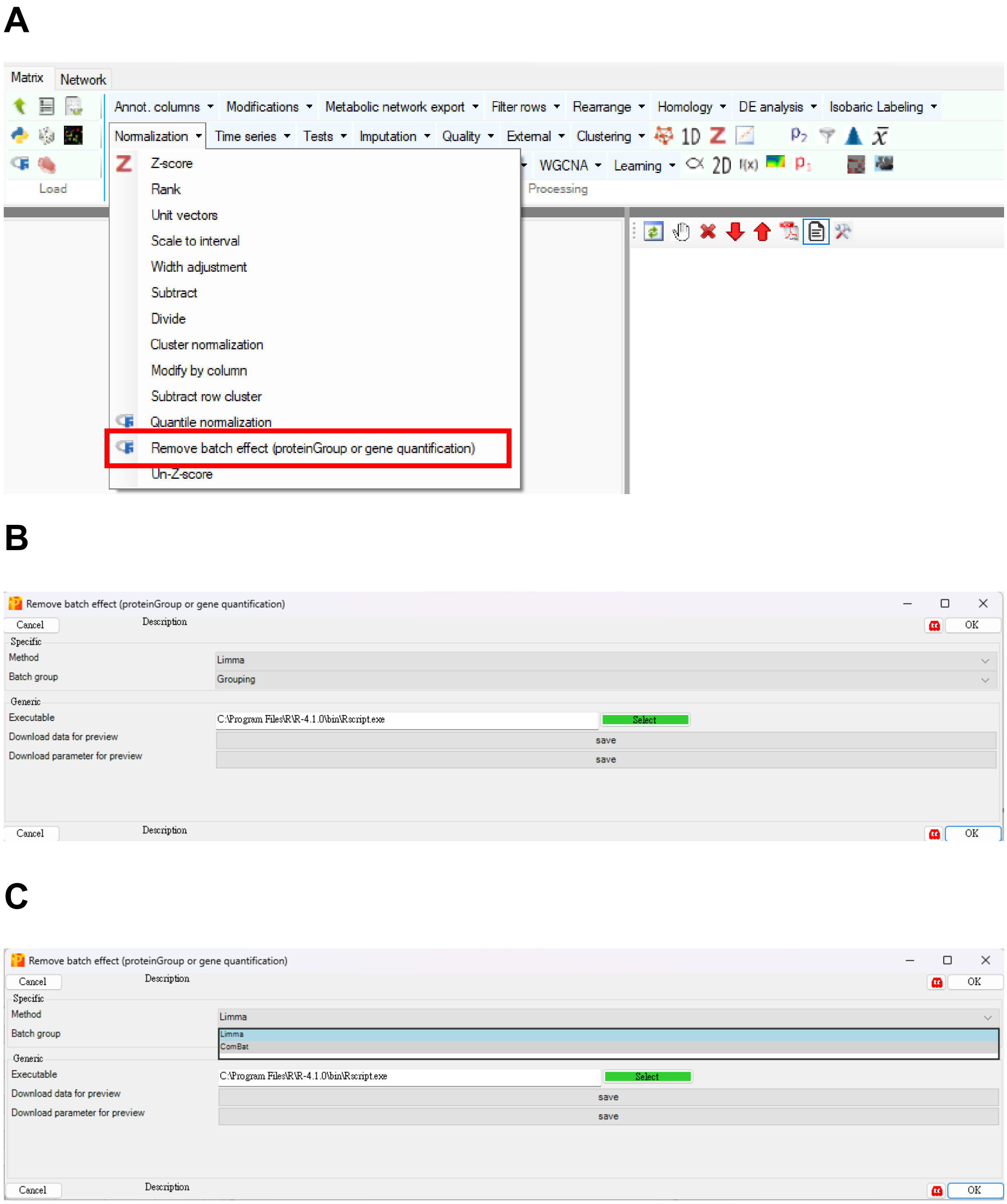

Fig. S3. The screenshots of the plug-in for Limma and ComBat normalization in Perseus software.

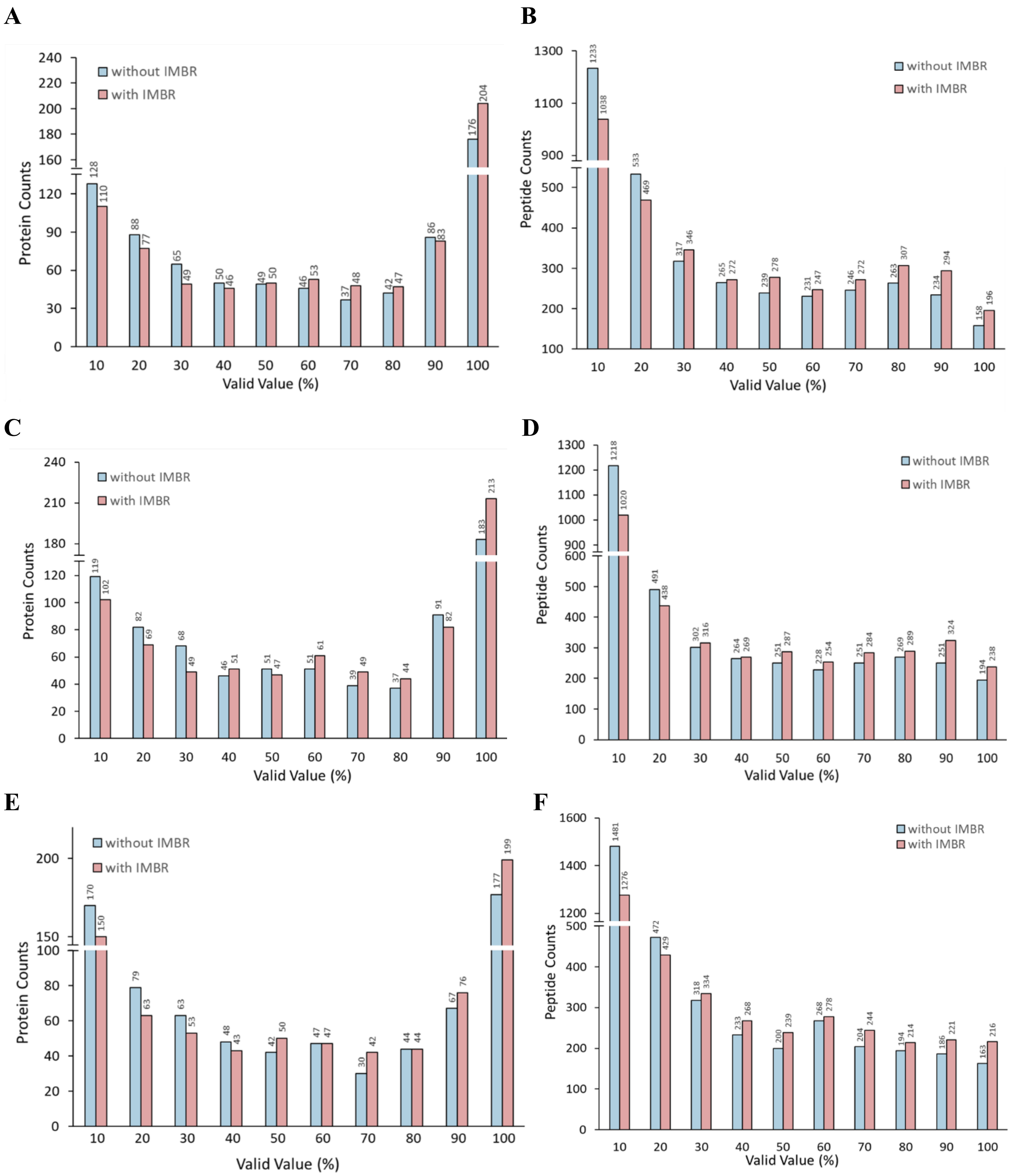

Fig. S5. Comparison of protein/peptide quantification with or without IMBR for SCoPE2 dataset. Pink and blue bars represent the number of quantified (**A**) proteins and (**B**) peptides with or without applying IMBR, respectively. IMBR significantly improved the number of quantified proteins and peptides in (**C**, **D**) macrophage cells and (**E**, **F**) monocytes. Using IMBR, a 16% and 24% increase was observed in proteins and peptides with less than 10% missing values across 11 TMT 11-plex batches. On the other hand, the number of proteins and peptides owning more than 90% missing values across 11 TMT sets drops 14% and 16, respectively.

A

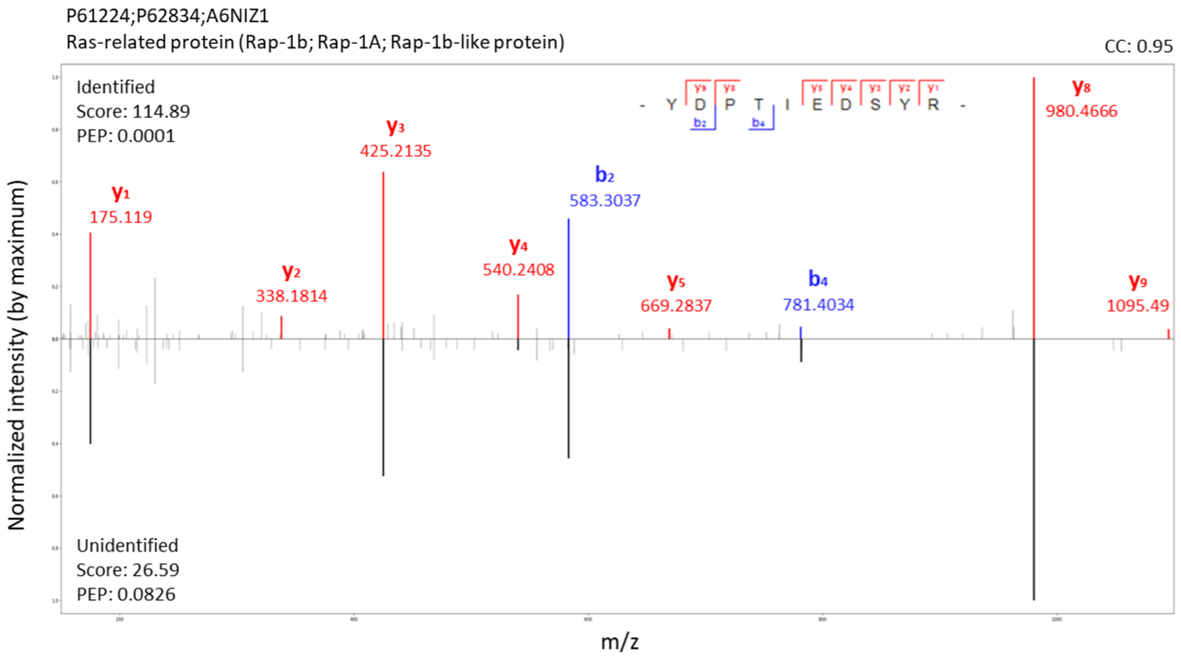
P61224;P62834;A6NIZ1
Ras-related protein (Rap-1b; Rap-1A; Rap-1b-like protein)
CC: 0.95
Identified
Score: 114.89
PEP: 0.0001
- Y D P T I E D S Y R -
y1 175.119
y2 338.1814
y3 425.2135
y4 540.2408
b2 583.3037
y5 669.2837
b4 781.4034
y8 980.4666
y9 1095.49
Unidentified
Score: 26.59
PEP: 0.0826
Normalized intensity (by maximum)
m/z

B

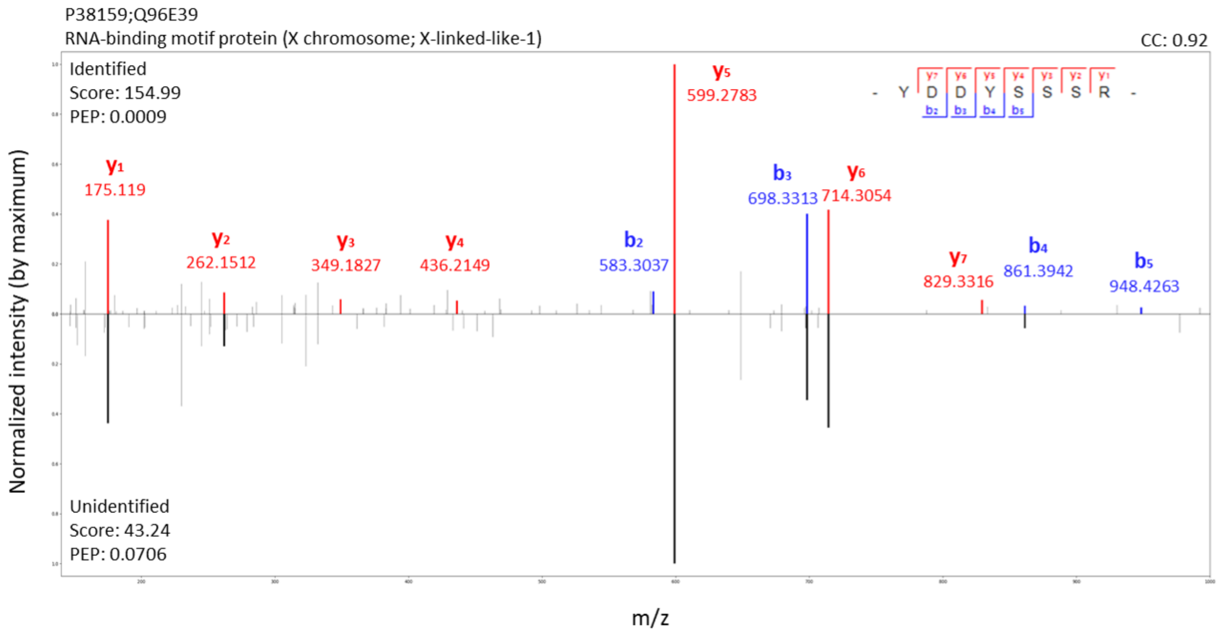
P38159;Q96E39
RNA-binding motif protein (X chromosome; X-linked-like-1)
CC: 0.92
Identified
Score: 154.99
PEP: 0.0009
- Y D D Y S S S R -
y1 175.119
y2 262.1512
y3 349.1827
y4 436.2149
b2 583.3037
y5 599.2783
b3 698.3313
y6 714.3054
y7 829.3316
b4 861.3942
b5 948.4263
Unidentified
Score: 43.24
PEP: 0.0706
Normalized intensity (by maximum)
m/z

C

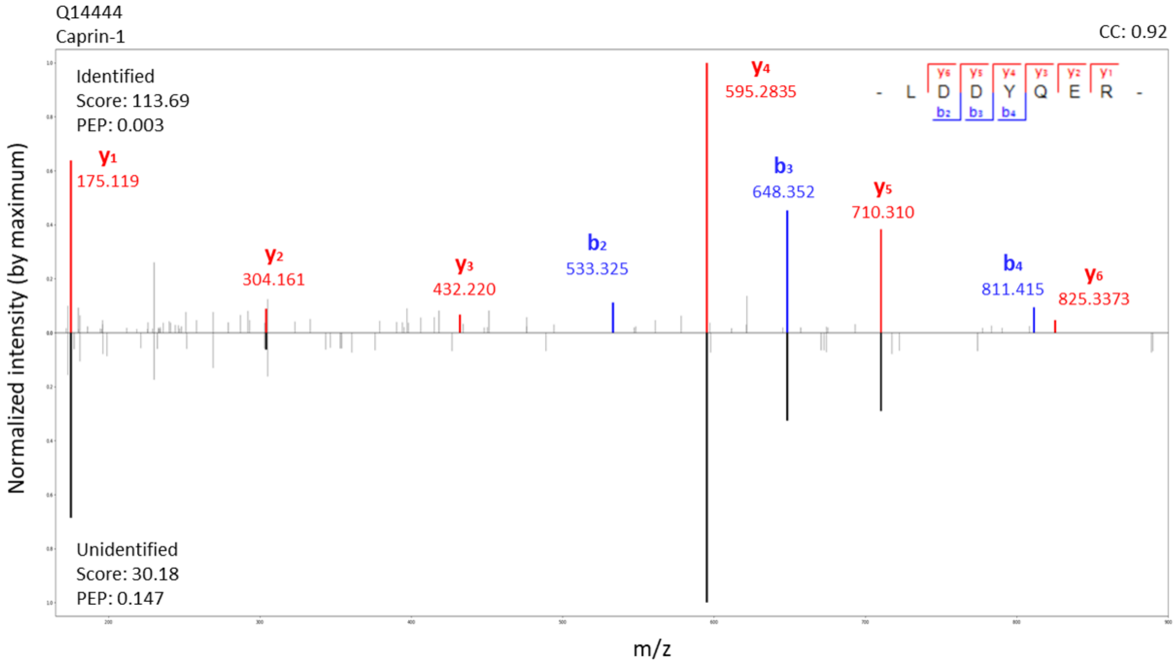

D

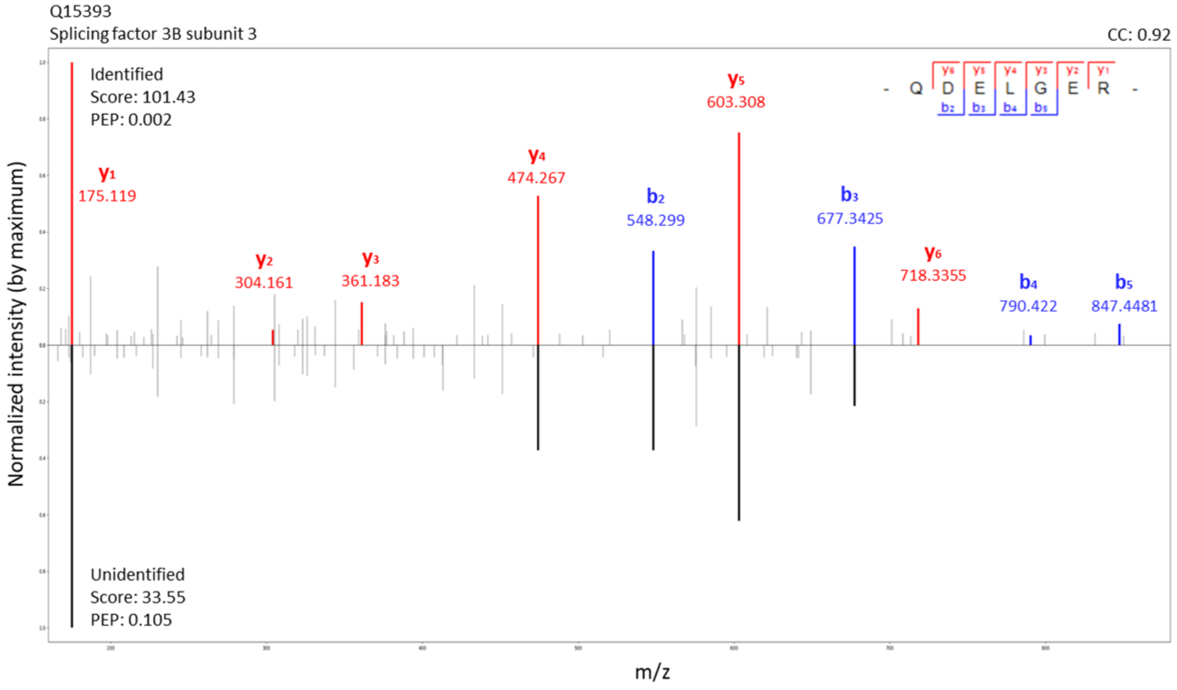

Fig. S6. Alignment of b/y-ions between identified and IMBR-rescued MS2 spectra. MS2 spectrum derived from (A) Ras-related protein, (B) RNA-binding motif protein, (C) Caprin-1, and (D) Splicing factor 3B subunit 3 are selected as examples. The upper panel shows the experimentally PSM with the peptide sequence, identification score, and PEP value. The corresponding bottom panel represents the matching spectra. Blue and red peaks represent the b and y ions, respectively. Pearson correlation coefficients (CC) between two MS2 spectra was all higher than 90%.

A

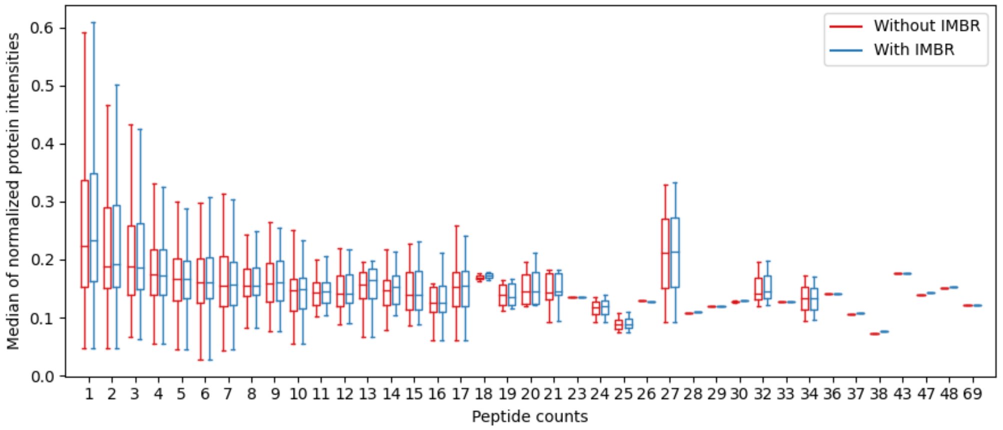

B

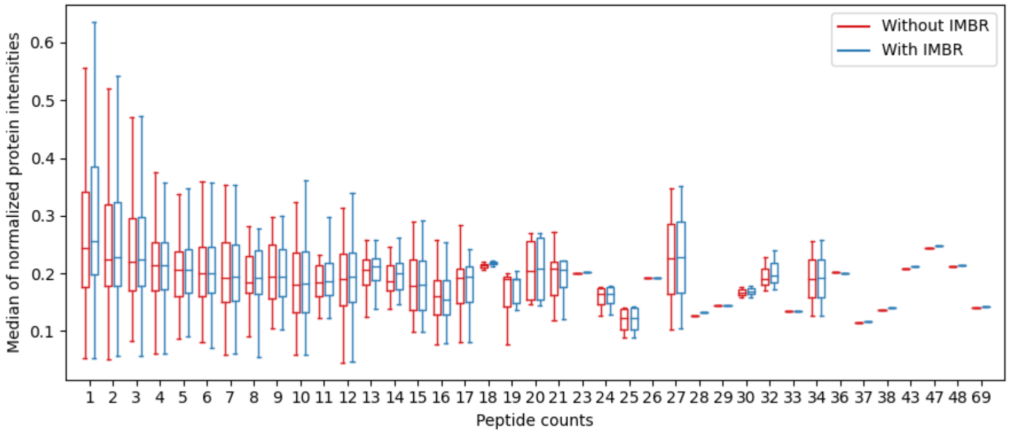

C

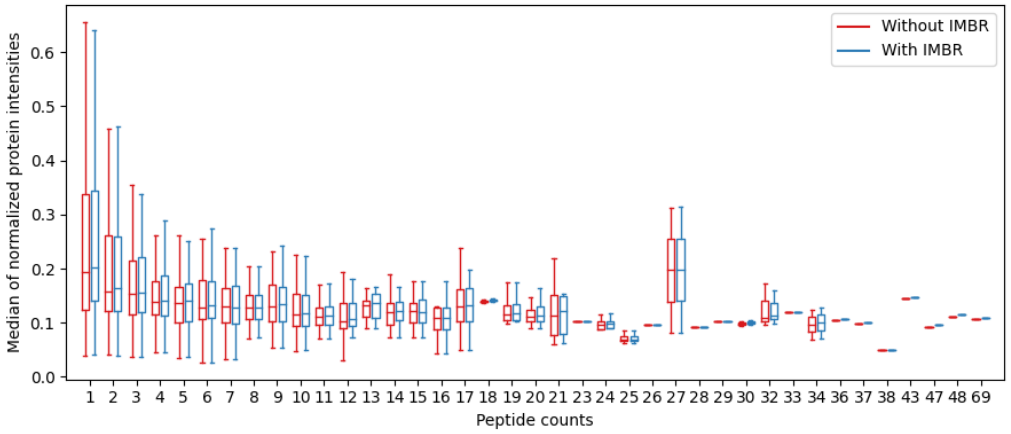

Fig. S7. Boxplots of normalized protein intensities with or without IMBR. Y-axis represents the median of protein intensities with PSM-level normalization, while x-axis shows the number of identified peptides of proteins. Red and blue boxes indicate the relative protein abundance quantified with or without using IMBR and the line inside box represents the median value. From (A) to (C) are the analysis results for all cell types, melanomas, and monocytes, respectively. All p-values between blue and red boxes are higher than 0.05.

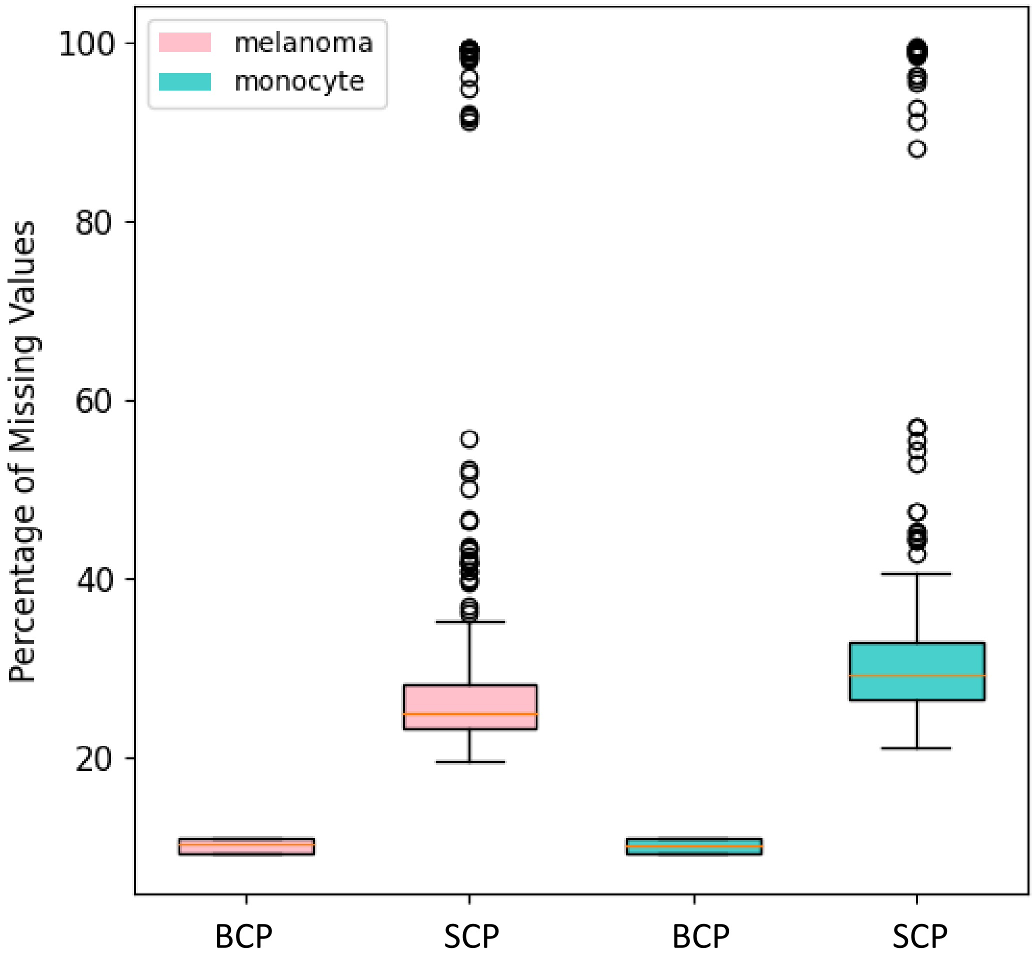

Fig. S8. Comparison of missing value content between SCP and BCP datasets. The pink and cyan boxes represent melanoma cells and monocytes, respectively. The median of each data group is presented by the red line. *P*-values for both cell types between BCP and SCP are 1.24e-65 and 5.37e-52, respectively.

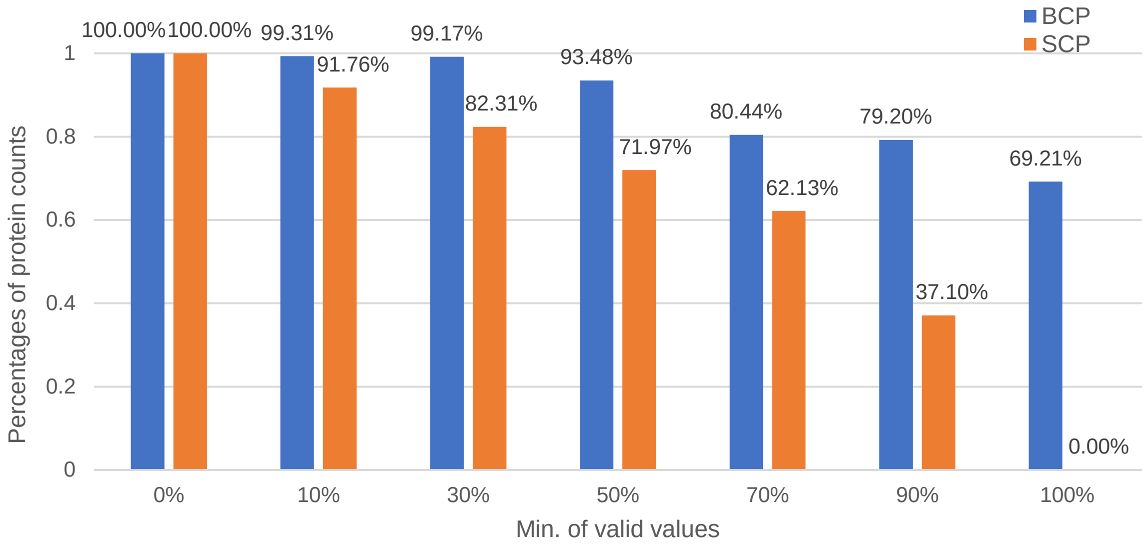

Fig. S9. The protein counts of BCP and SCP dataset with different missing value filtering thresholds. Each bar represents the remaining proteins after applying the filtering based on minimum of valid values of a protein. Blue bar means the protein counts from BCP dataset and orange bar is the protein counts from SCP dataset. Values on the top of bars indicate the percentage of remaining proteins against total proteins.

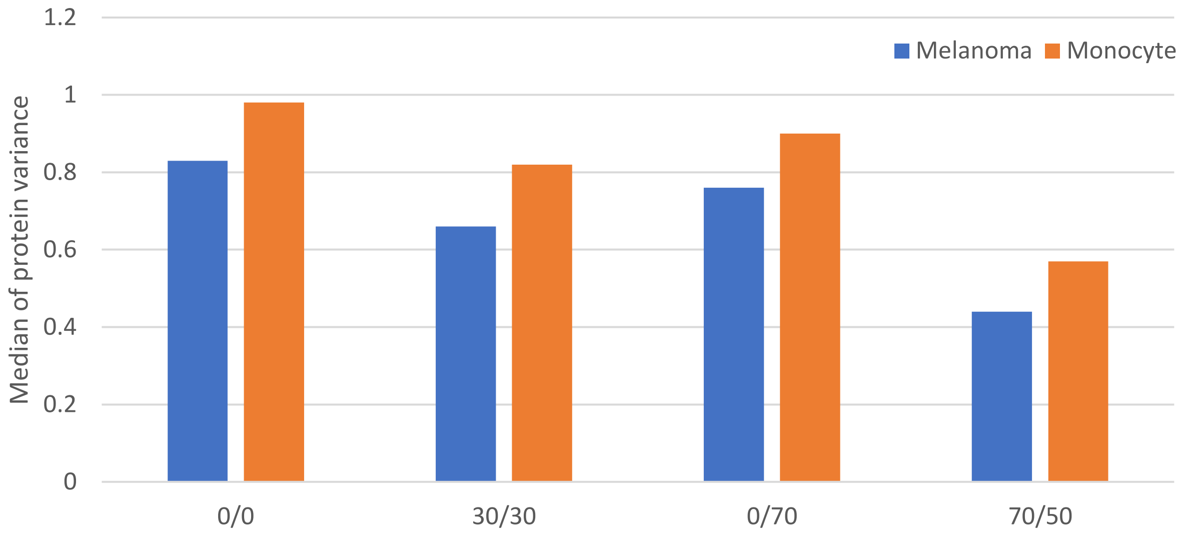

Fig. S10. The protein variance between different missing value filtering strategies. Blue and orange bars represent the median of protein variances for melanoma cells and monocytes with different quantification control filtering. The numbers at x-axis indicate the minimum valid values of a cell and protein, such as 70/50 means only the cells containing ≥ 70% and proteins owning ≥ 50% valid values are kept.

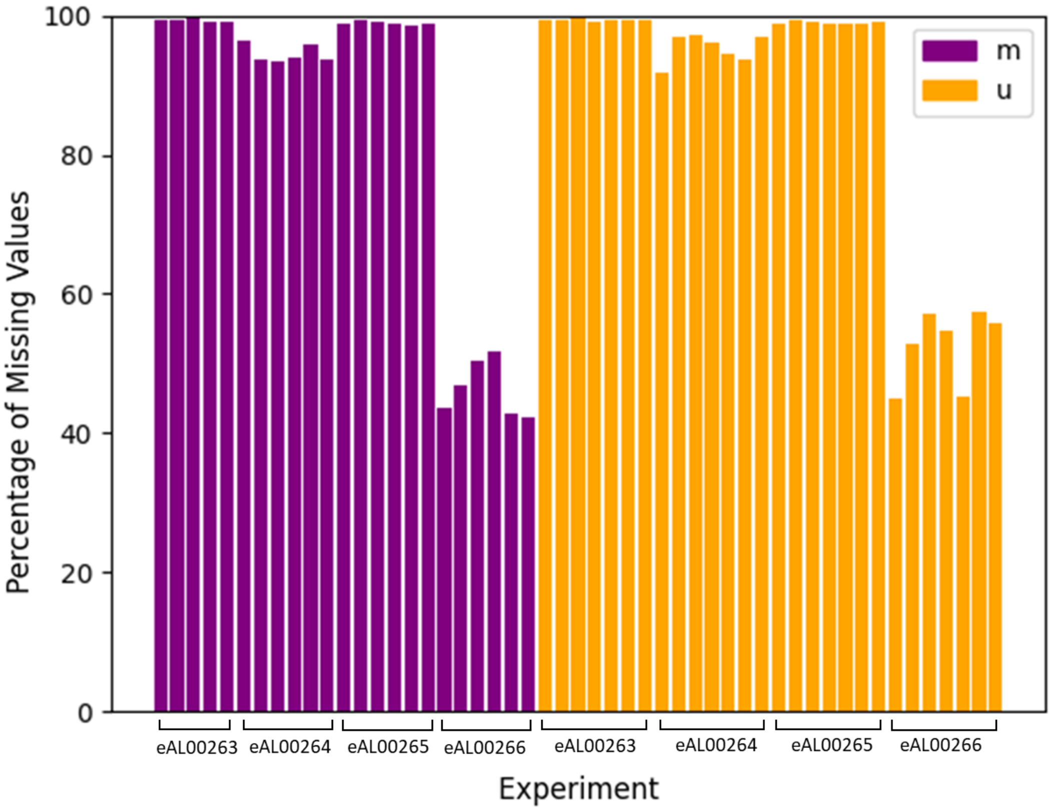

Fig. S11. Three TMT batches containing high proportion of missing values. Purple and yellow bars represent the percentage of missing values for melanoma and monocyte in three TMT18pro batches. eAL00266 is used as the reference for the comparison with the other three TMT batches - eAL00263 to eAL00265.

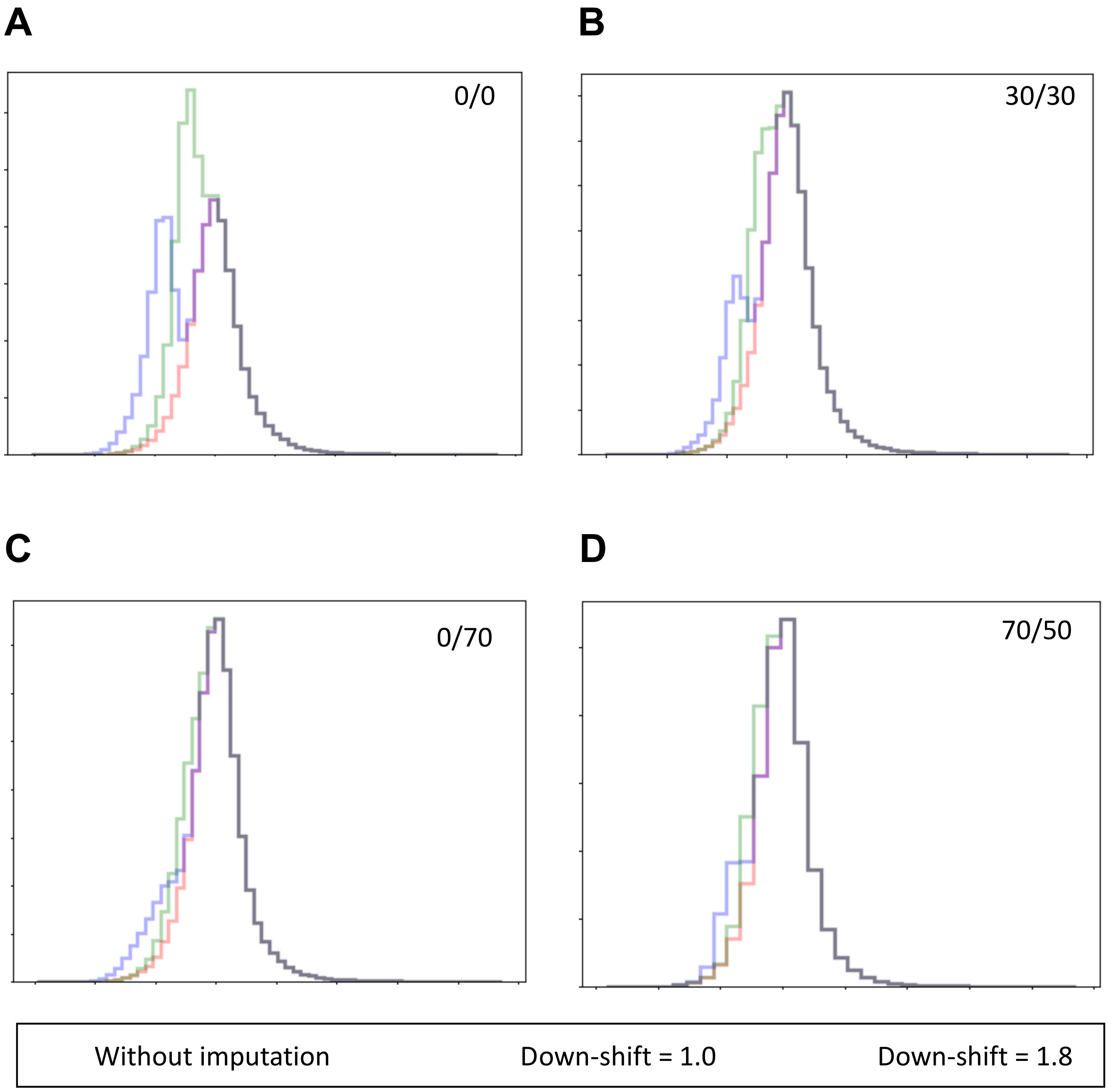

Fig. S12. The data distribution with and without imputation using the down-shift from 1.0 and 1.8. The pink lines represent the original data distribution. The light blue and light green lines show the data distribution of using imputation with down-shift as 1.8 and 1.0, respectively. From panel (A) to (D) are for four quantification control filtering – 0/0, 30/30, 0/70 and 70/50.

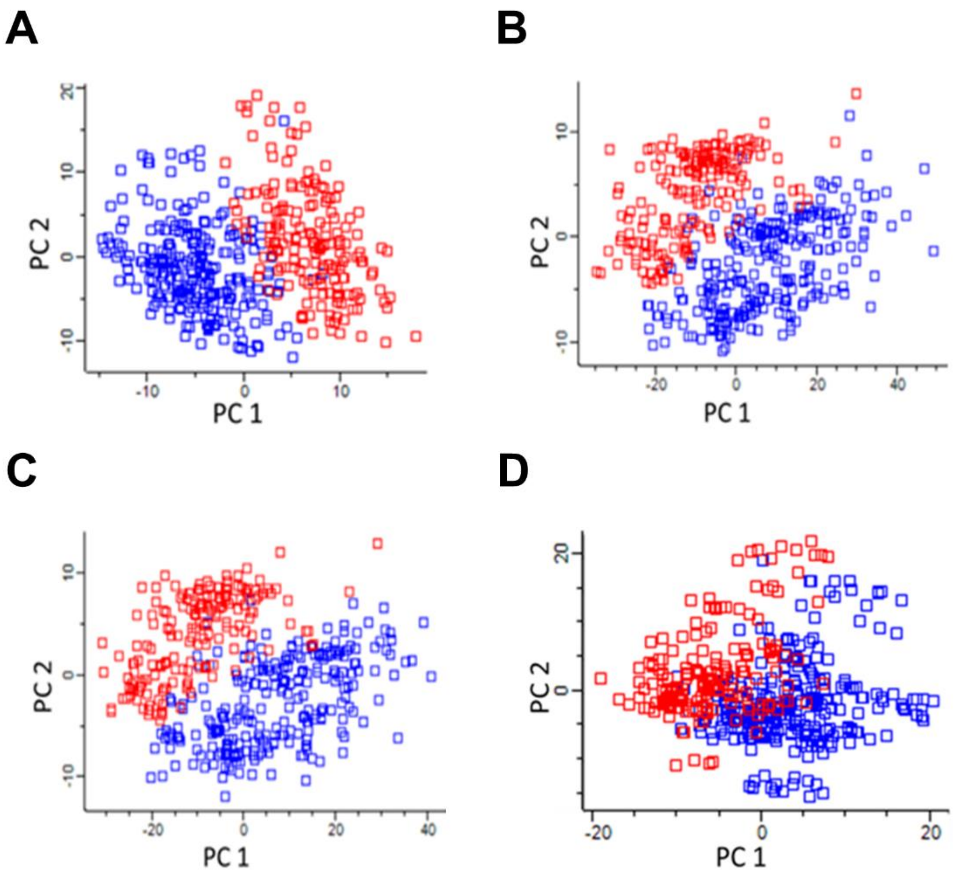

Fig. S13. PCA for the dataset with PSM-level, protein-level normalization, or without normalization. Red squares indicate the samples from melanoma cells while blue squares are from monocytes. The SCP dataset was processed with (A) PSM-level normalization, (B) Limma normalization, (C) ComBat normalization, and (D) without normalization.

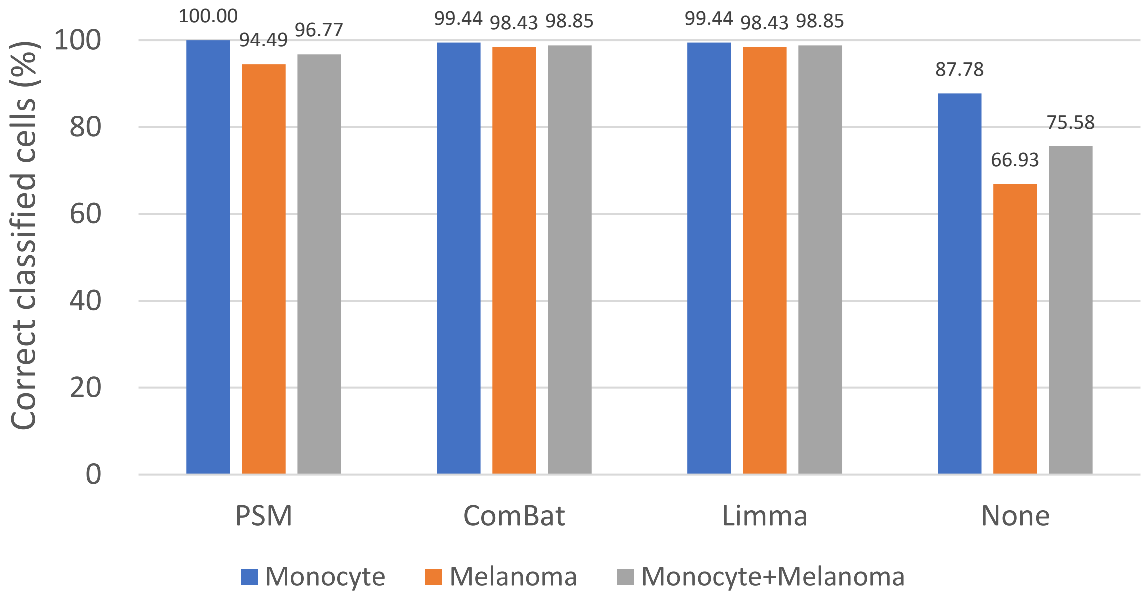

Fig. S14. Comparison of sample clustering using different normalization strategies. Y-axis means the data clusters from KMeans clustering matched the experimental setting. Blue, orange, and grey bars represent the results from monocyte, melanoma and the combination of two cell types. PSM means the dataset was normalized by PSM-level ratio weighted normalization and Combat and Limma are normalized at protein level. None represents the dataset was not subjected into any normalization.

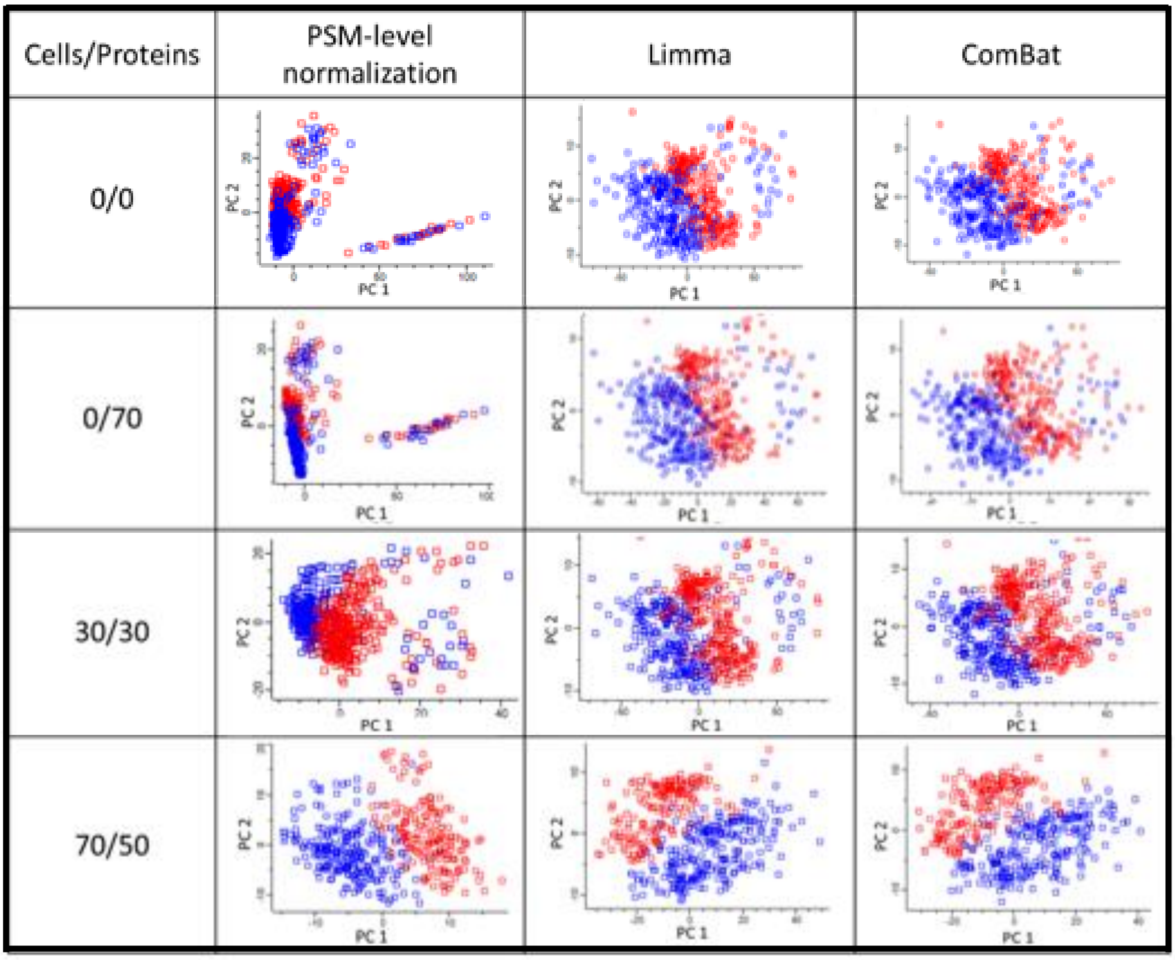

Fig. S15. PCA plots of SCP dataset with Limma, ComBat, or PSM-level normalization under different quantification control conditions. In the first column, the values before and after slash mean the minimum percentages of valid values in a cell and a protein, respectively. Red squares represent the melanoma samples and blue squares are the samples from monocyte cells. From the left to right columns show the PCA results with PSM-level normalization, Limma, and Combat normalization.

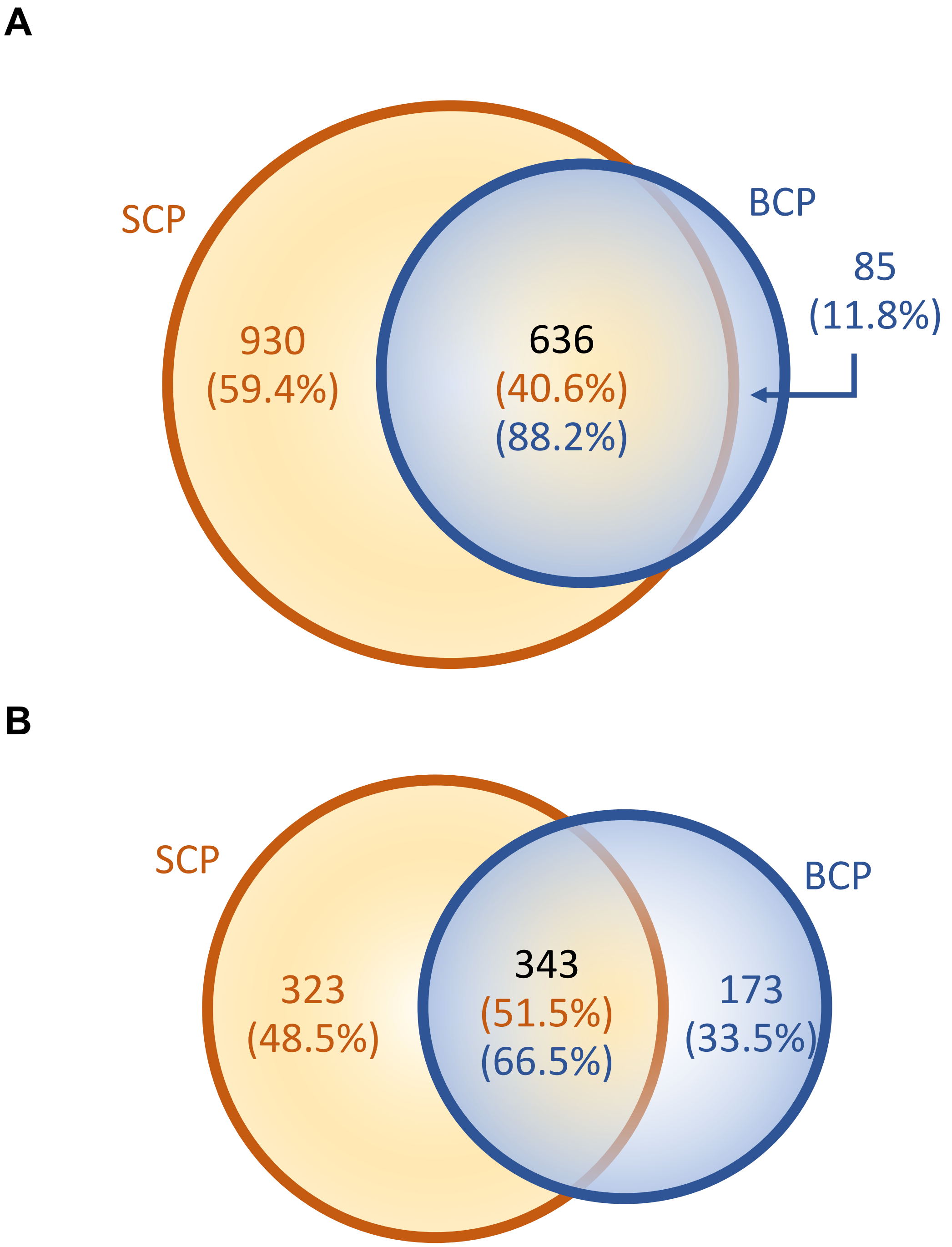

Fig. S16. Overlap of (A) all identified proteins and (B) differentially expressed proteins (DEPs) between SCP and BCP datasets. The brown circles and numbers are for the proteins in SCP and blue ones are for the proteins in BCP. The DEPs are detected by applying data filtering of 70/50.

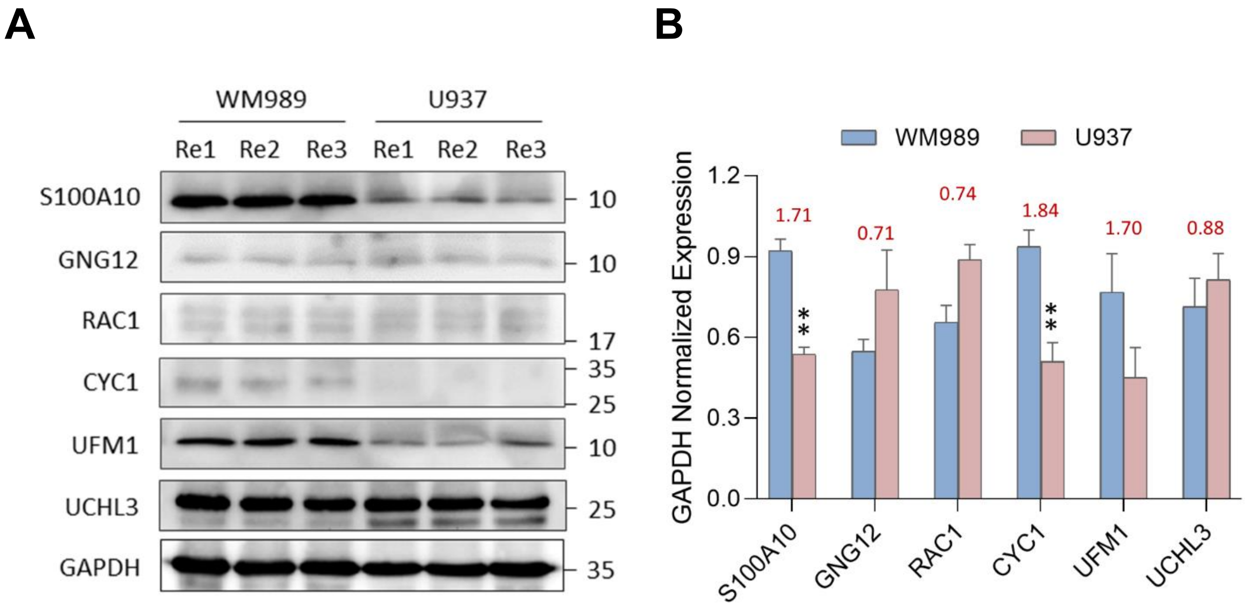

Fig. S17. Immunoblotting analysis of 6 selected DEPs with equal protein inputs. Equal protein amounts from melanoma or monocyte cells were loaded for SDS-PAGE analysis followed by western blotting. (A) Relative protein abundance of 6 selected targets were analyzed using western blotting. (B) Quantification of western blotting results using Image J. Error bars indicate ± S.D.E. ** means p-value ≤ 0.01. Values (red) indicated the protein expression level relative to that expressed in U937 cells.

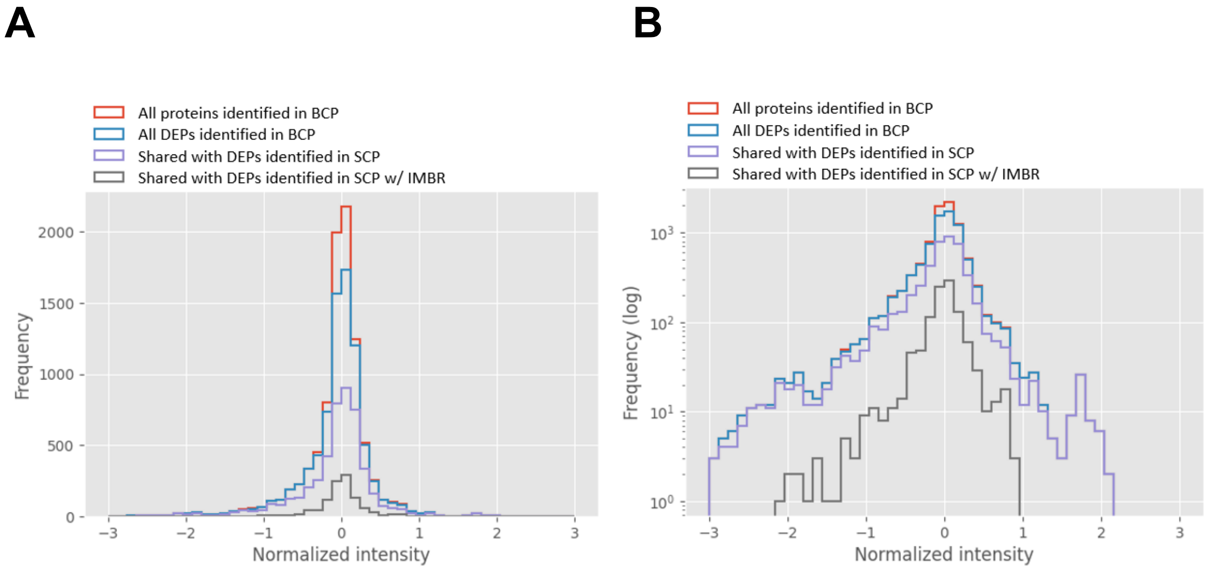

Fig. S18. Distributions of protein abundance from the DEPs identified in SCP and all identified proteins and DEPs from BCP. The difference between (A) and (B) is the y-axis scale that in (A) is original protein abundance while is logarithm transformed in (B). Red, blue, purple, and black lines represent the distribution of protein abundance from all identified proteins in BCP, DEPs identified in BCP, DEPs commonly determined in SCP w/ and w/o IMBR, respectively. *P*-values of the pairwise Mann-Whitney U tests are all larger than 0.05.

Table S2. The *q*-values of the 6 selected proteins for the validation in SCP and BCP dataset

**Proteins**	**SCP with IMBR**	**SCP without IMBR**	**BCP**
CYC1	$9.07e^{-10}$	Filtered out*	Not identified**
S100A10	$1.94e^{-7}$	0.063	$1.73e^{-12}$
RAC1	$4.76e^{-4}$	0.105	$1.20e^{-12}$
GNG12	$3.77e^{-7}$	0.048	$2.46e^{-7}$
UCHL3	$1.61e^{-3}$	Filtered out	Not identified
UFM1	$8.87e^{-8}$	0.117	Not identified

*The protein can be identified but is filtered out by applying data filtering.

**The protein cannot be not identified from the dataset.
