# Supplementary material for: Quantification Quality Control Emerges as a Crucial Factor to Enhance Single-Cell Proteomics Data Analysis
Source: Mol Cell Proteomics. 2024 Apr 15;23(5):100768. doi: 10.1016/j.mcpro.2024.100768 (PMC11103571; doi:10.1016/j.mcpro.2024.100768)

O94979
Protein transport protein Sec31A

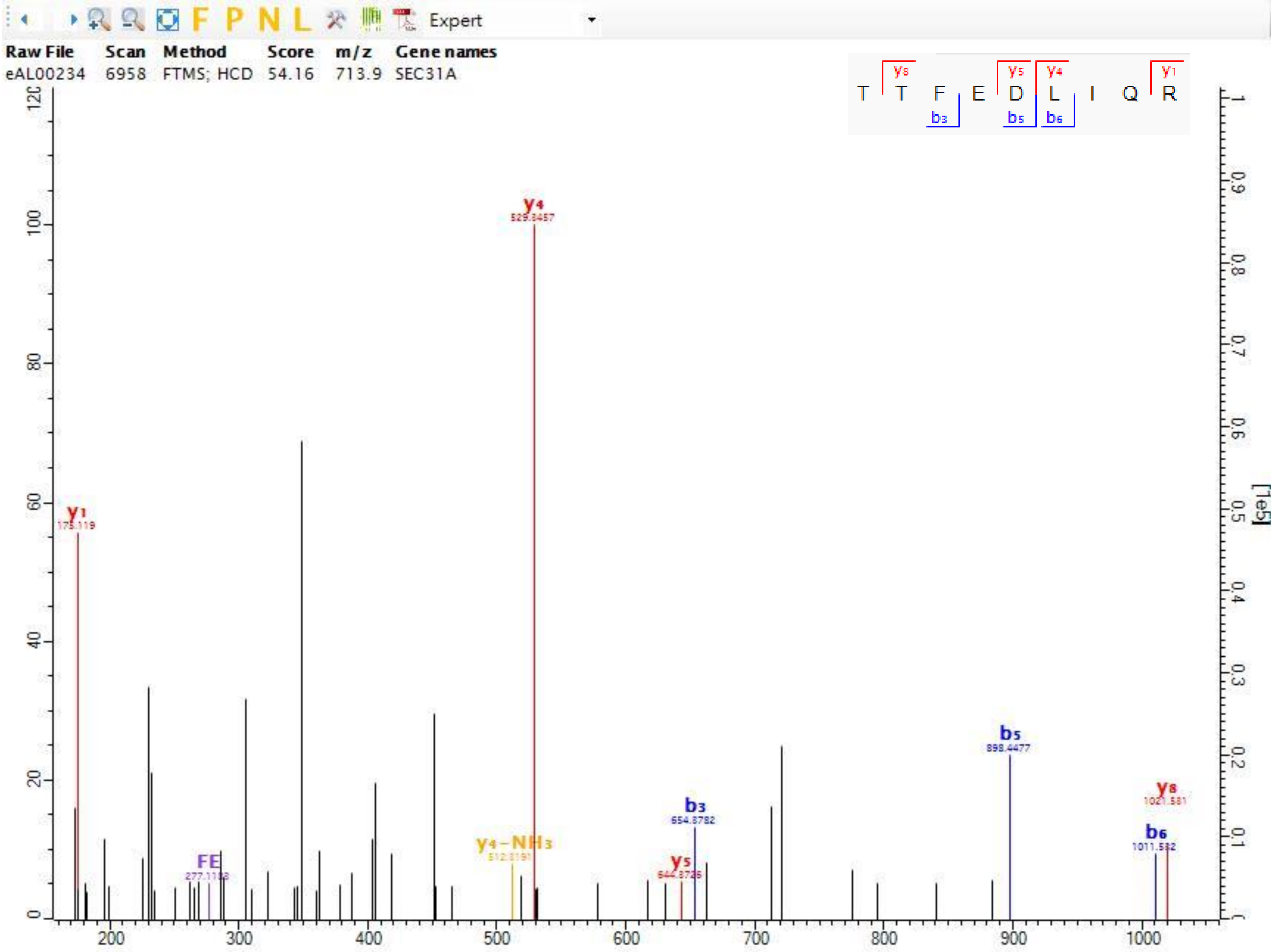

P07988
Pulmonary surfactant-associated protein B

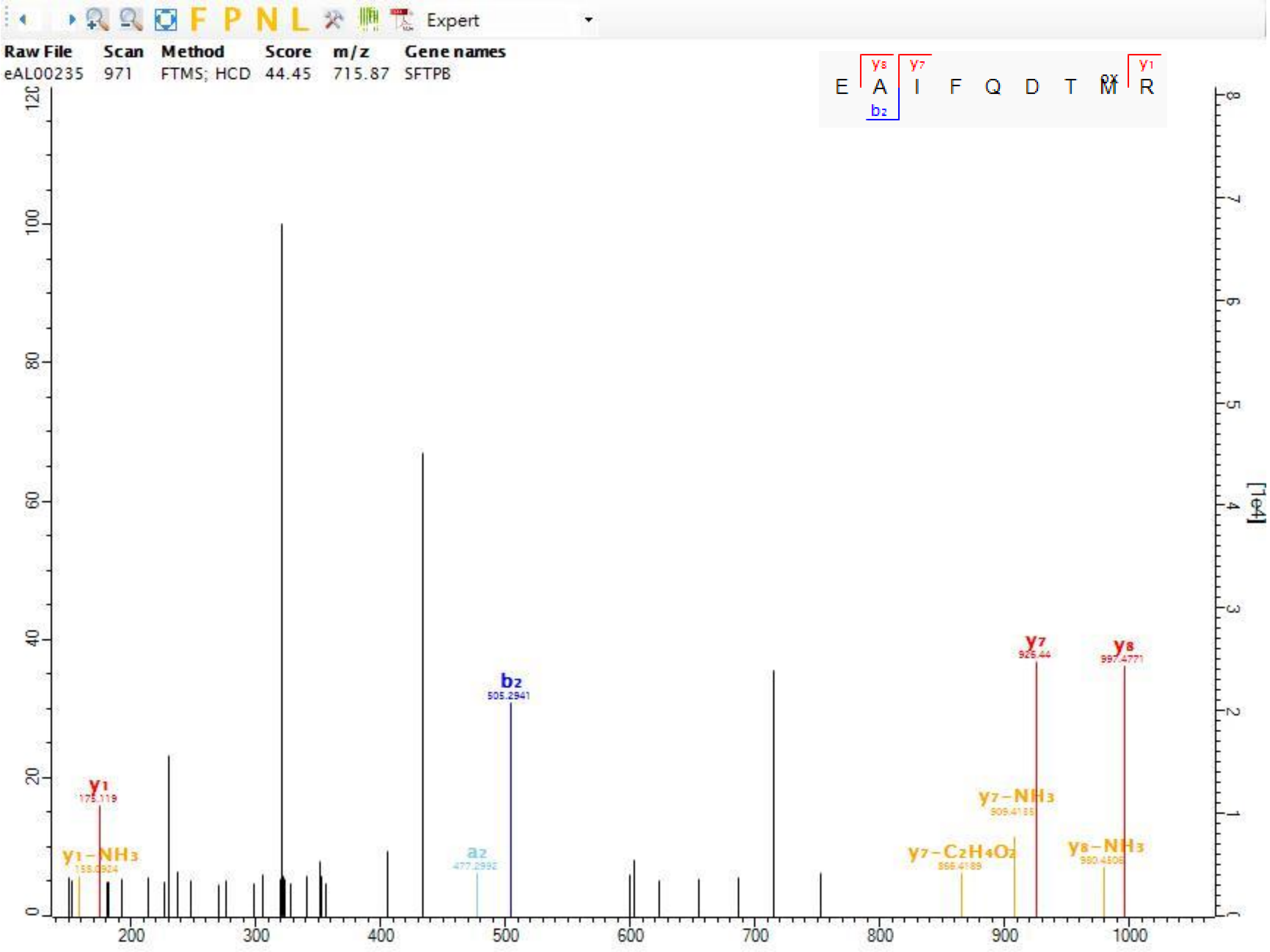

P11234
Ras-related protein Ral-B

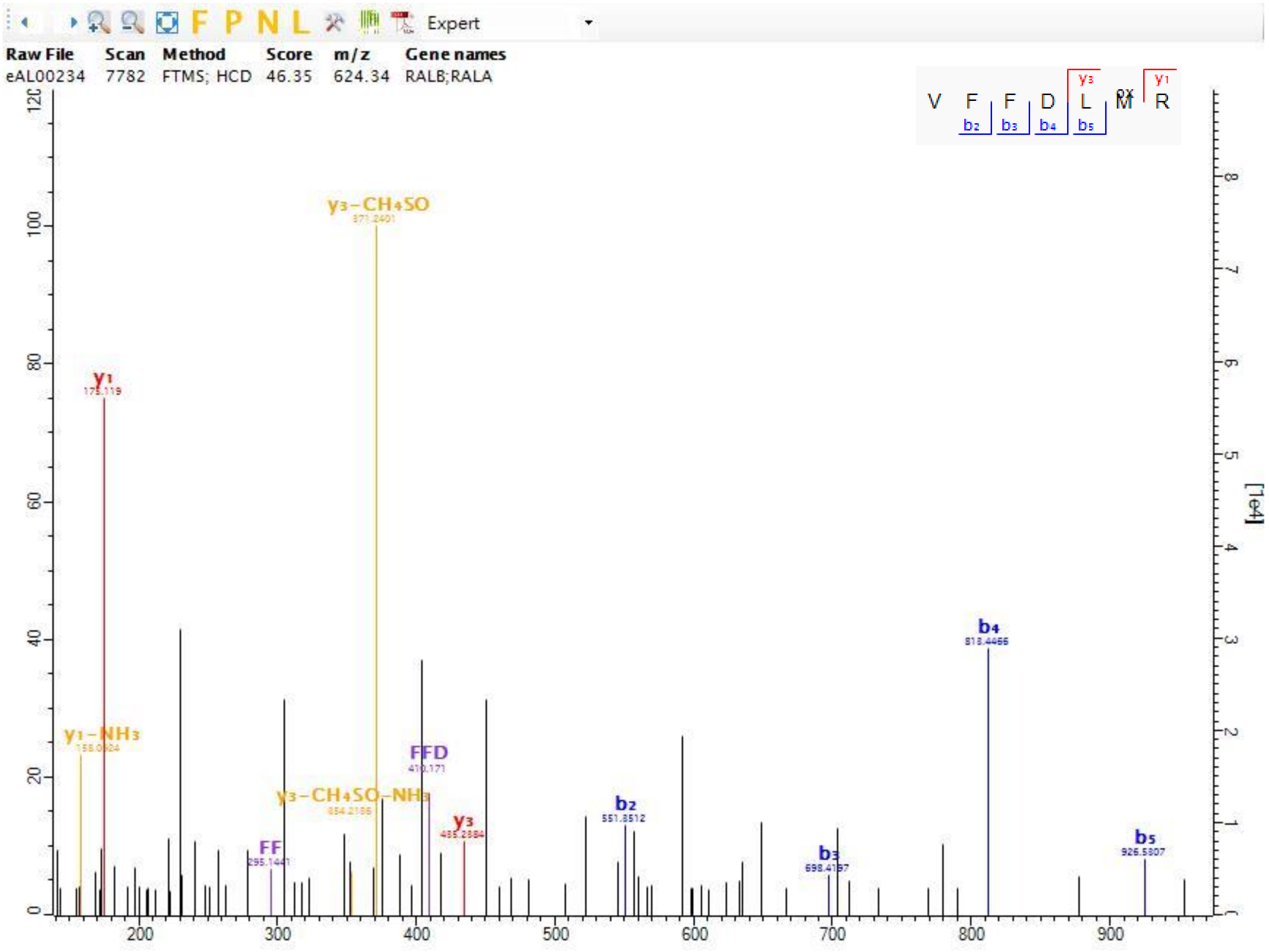

P60602
Reactive oxygen species modulator 1

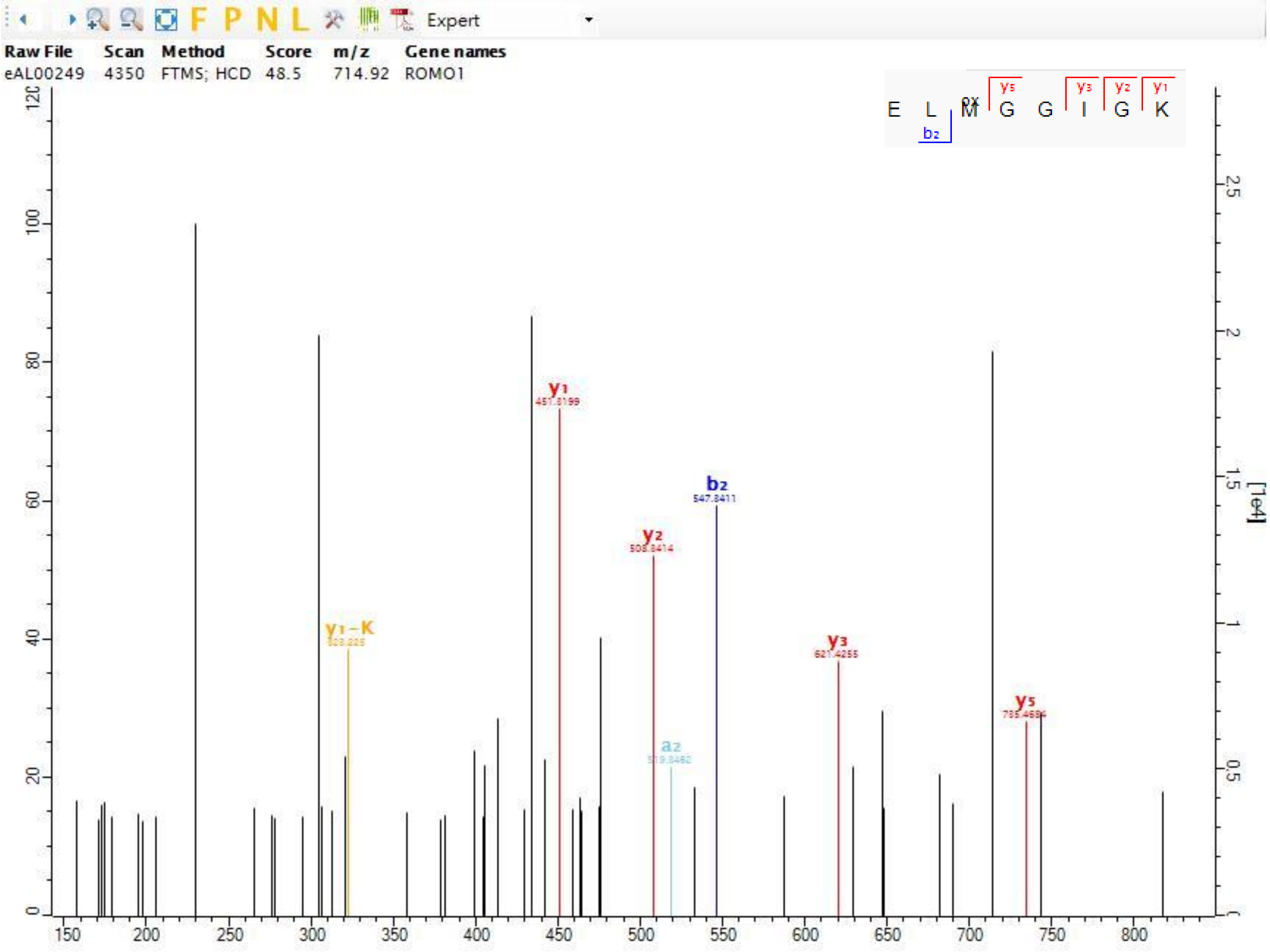

Q05135
Replication protein E1

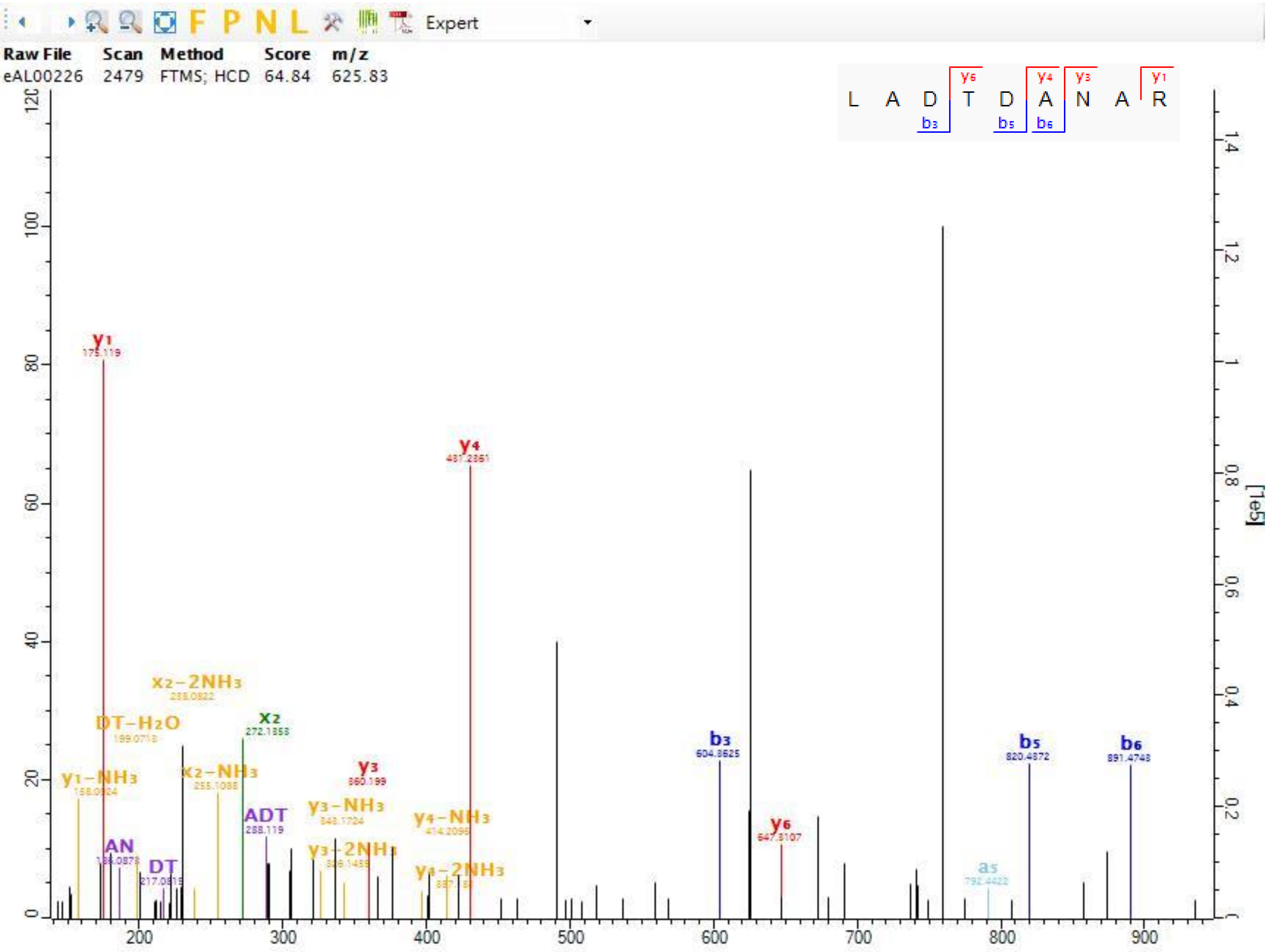

Q12962
Transcription initiation factor TFIID subunit 10

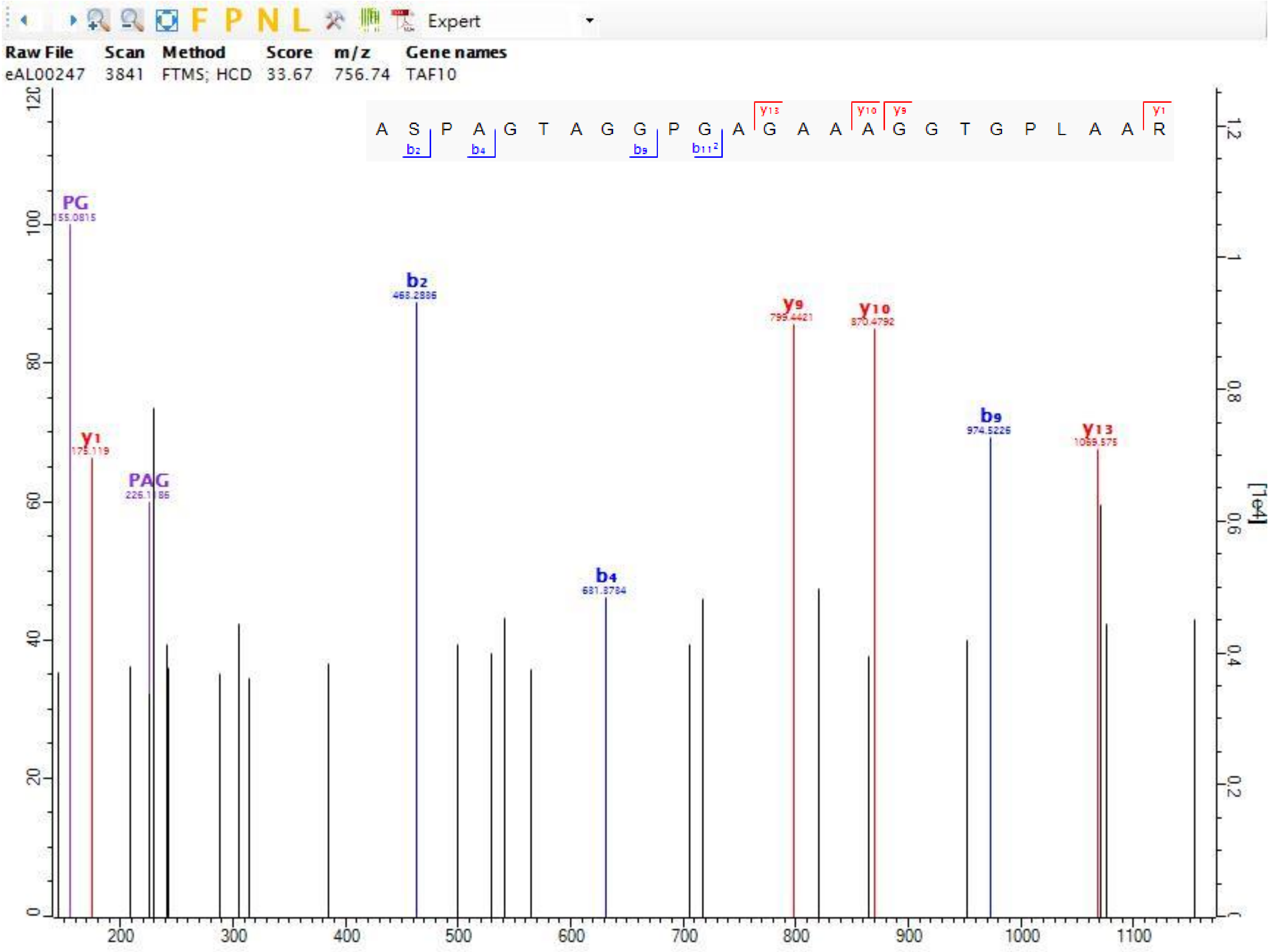

Q14315
Filamin-C

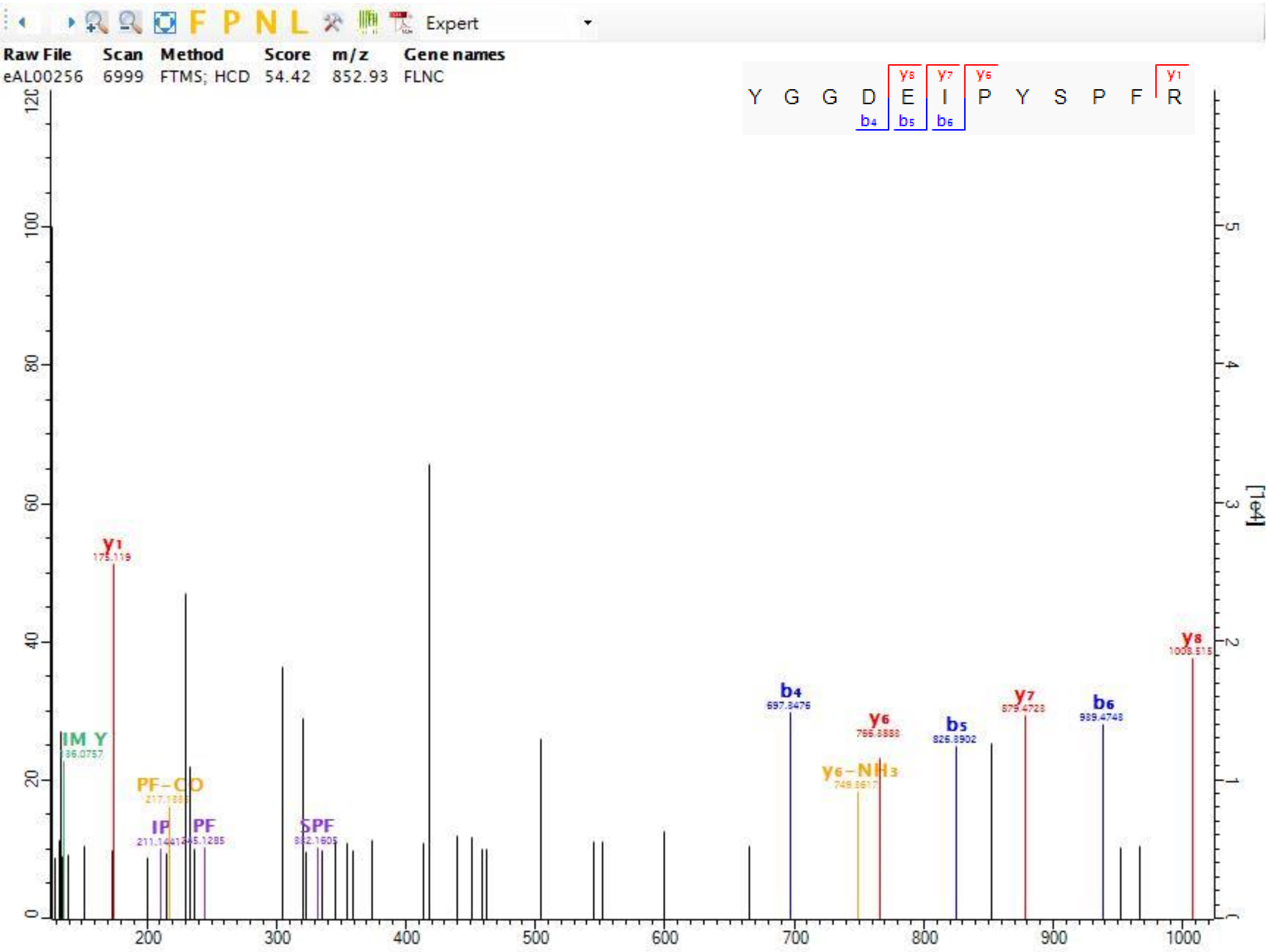

Q14353
Guanidinoacetate N-methyltransferase

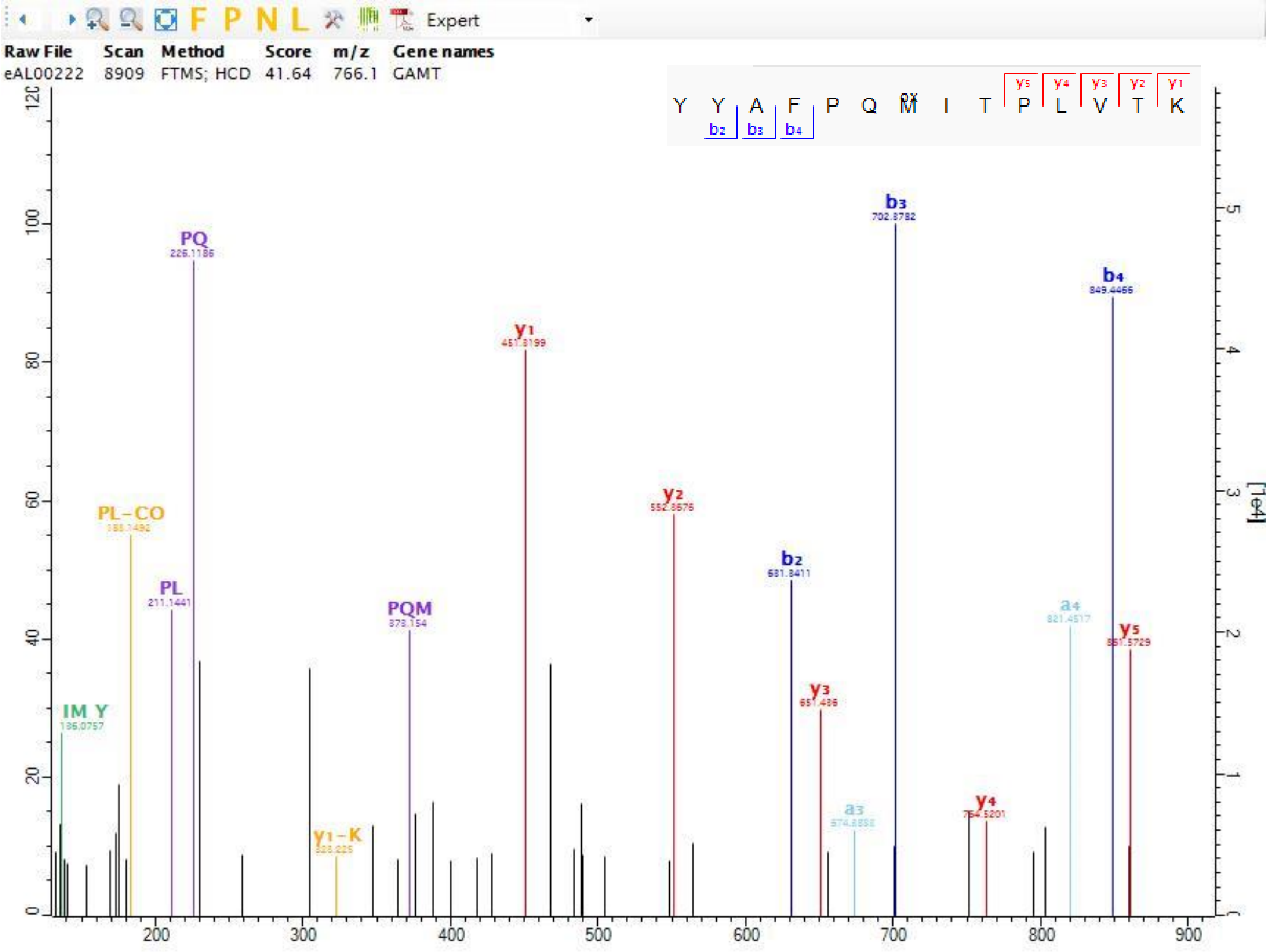

Q15286
Ras-related protein Rab-35

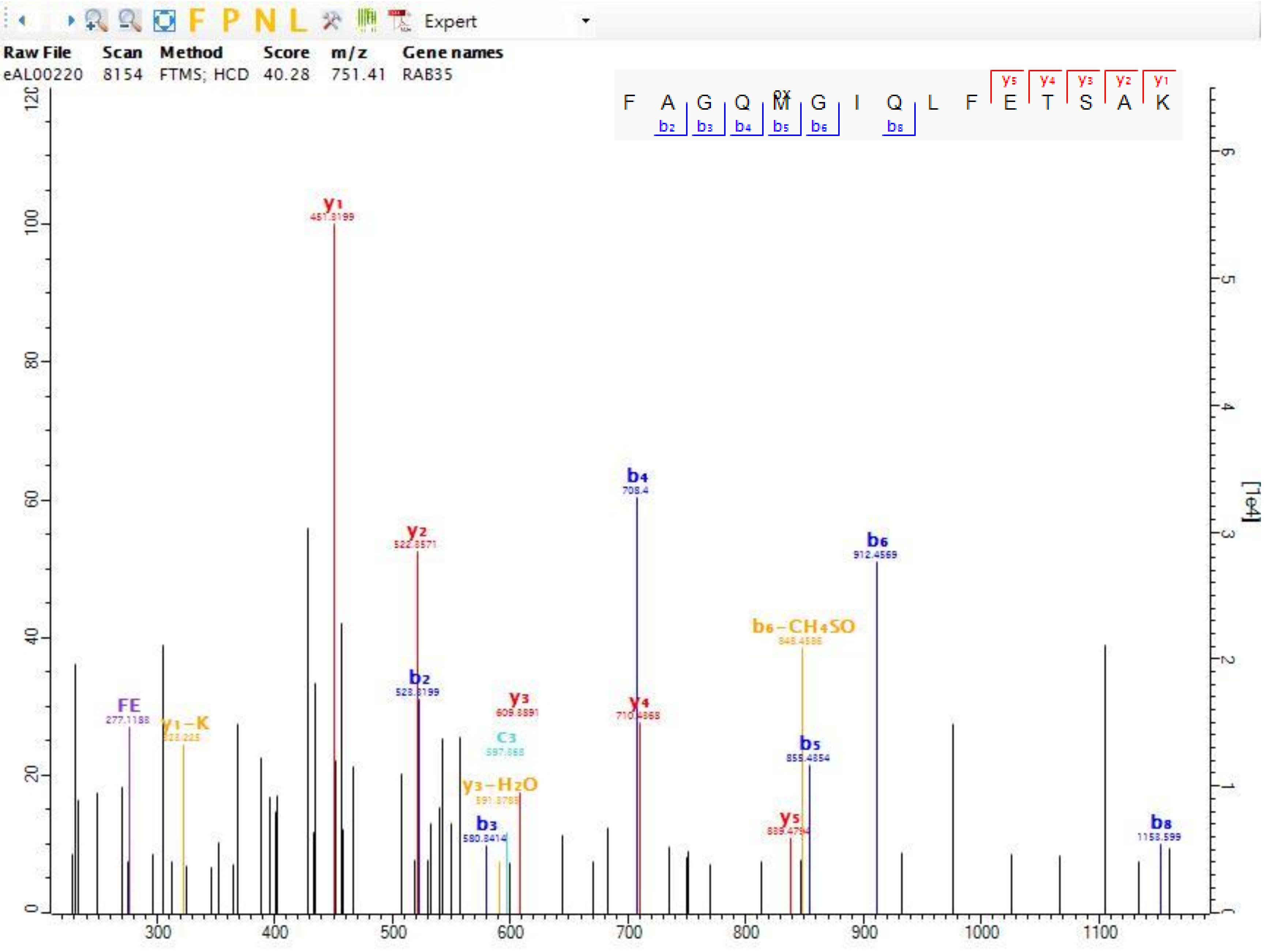

Q15417
Calponin-3

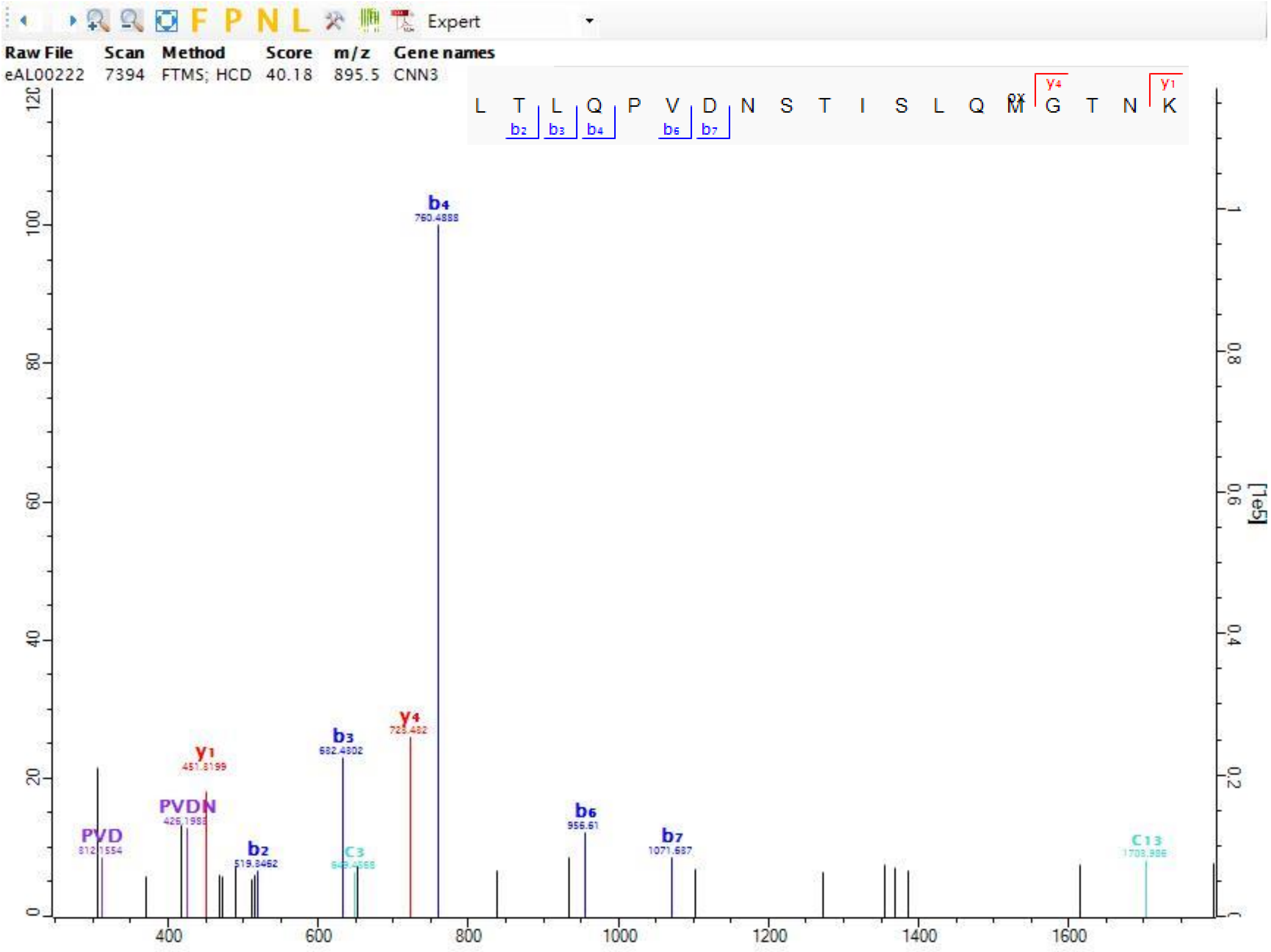

Q4VNC1
Probable cation-transporting ATPase 13A4

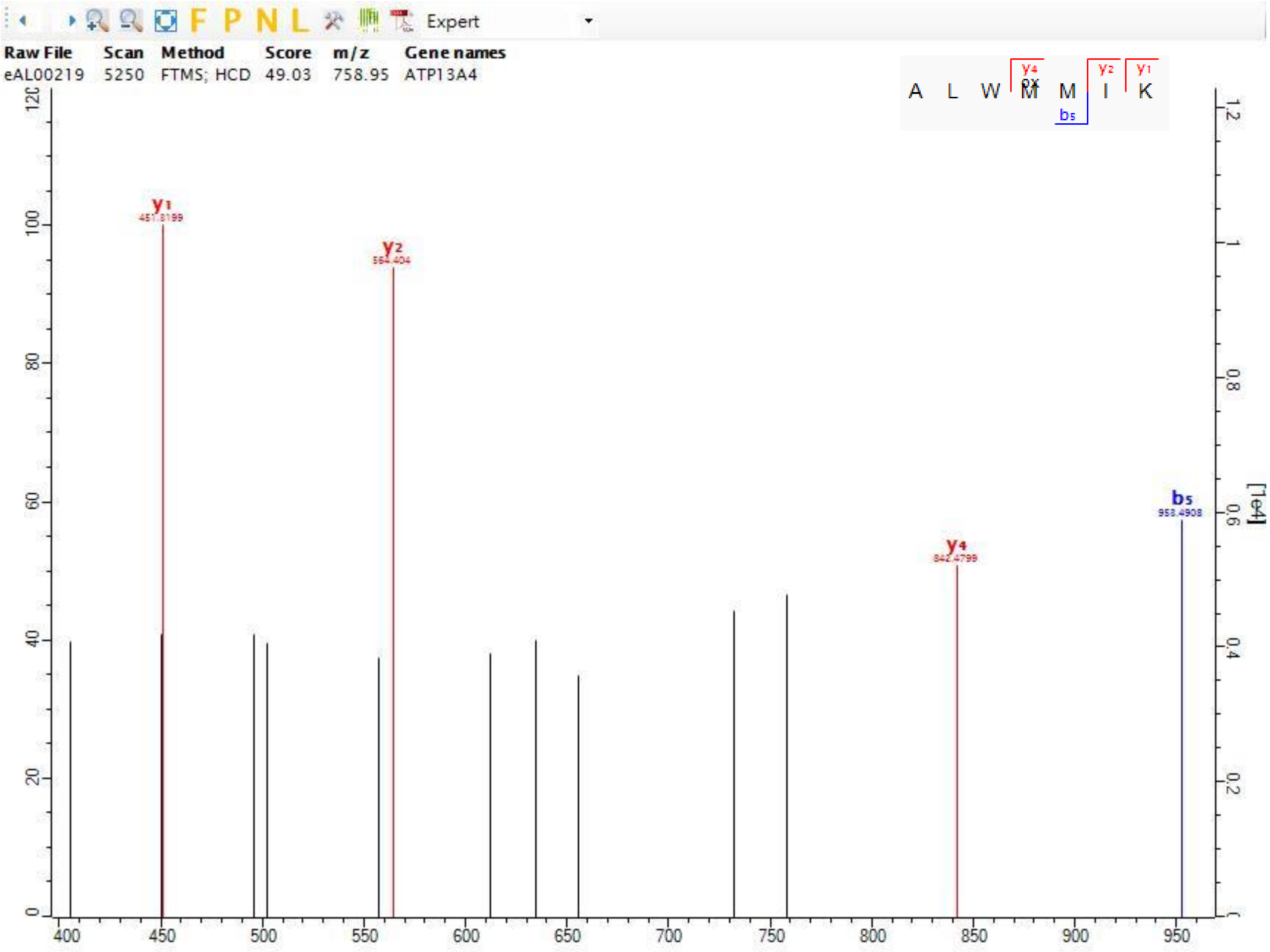

Q6ZNC8
Lysophospholipid
acyltransferase 1

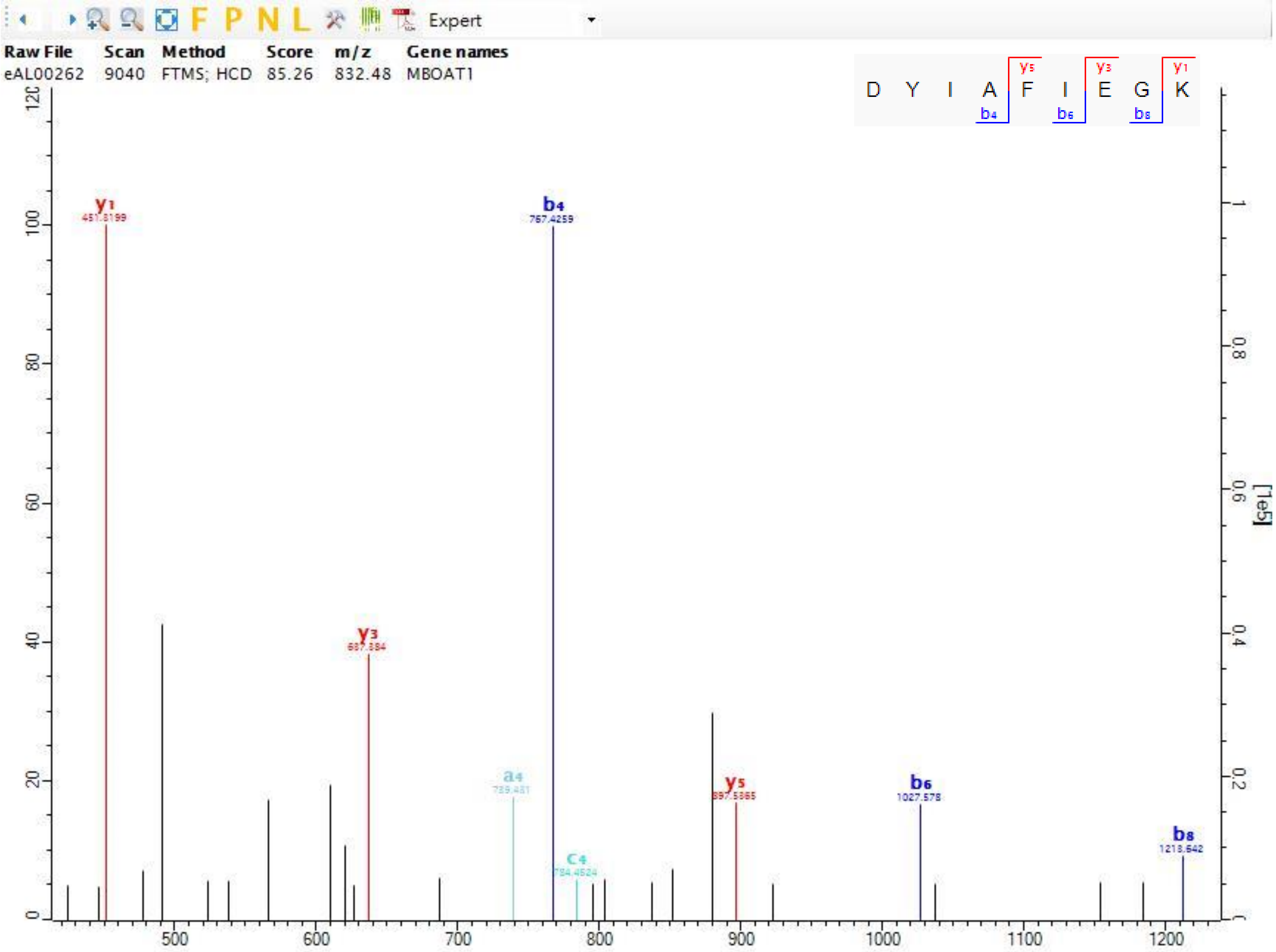

Q8IYB3
Serine/arginine repetitive matrix protein 1

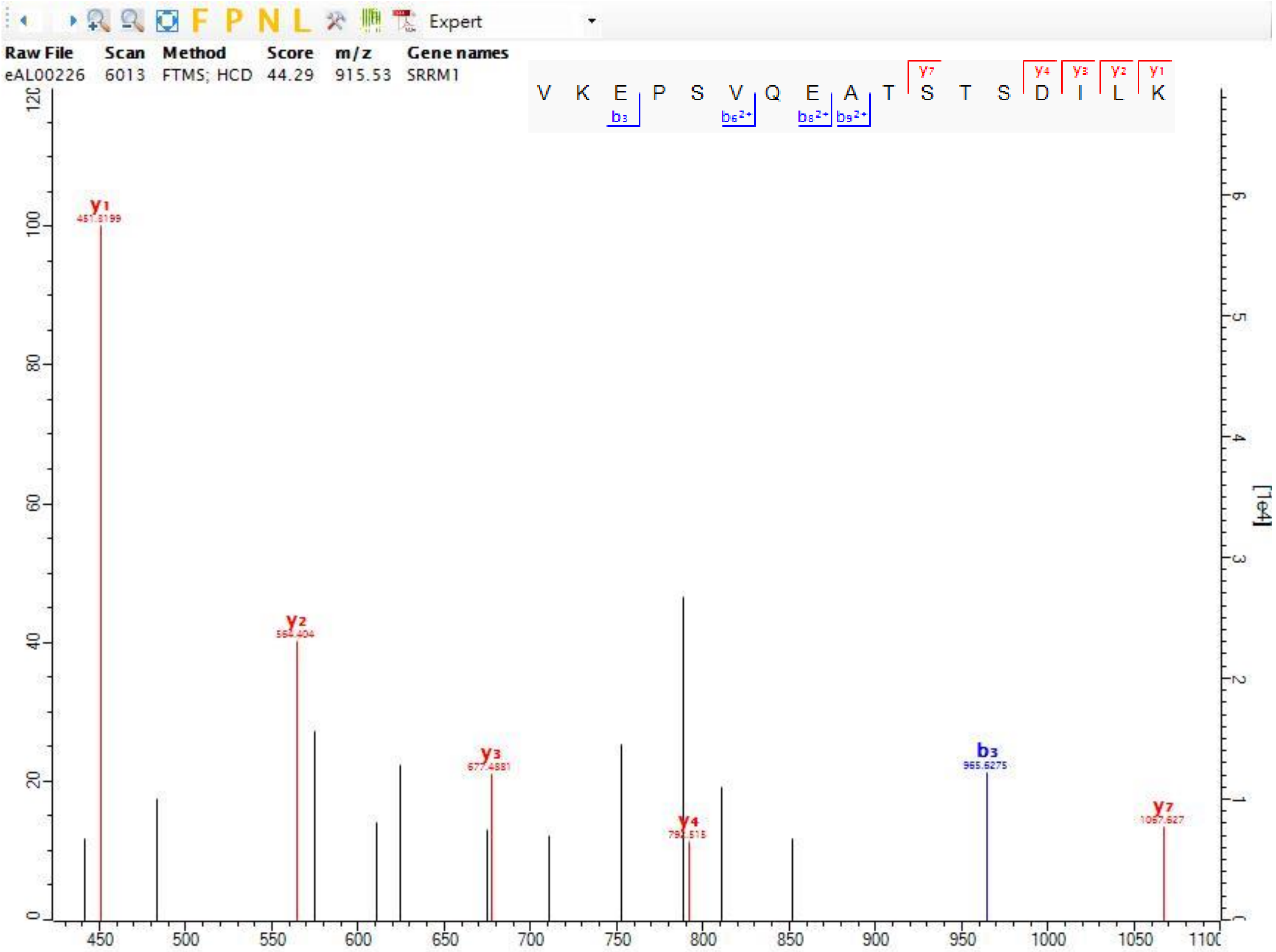

Q8QMQ7
Thymidylate
kinase

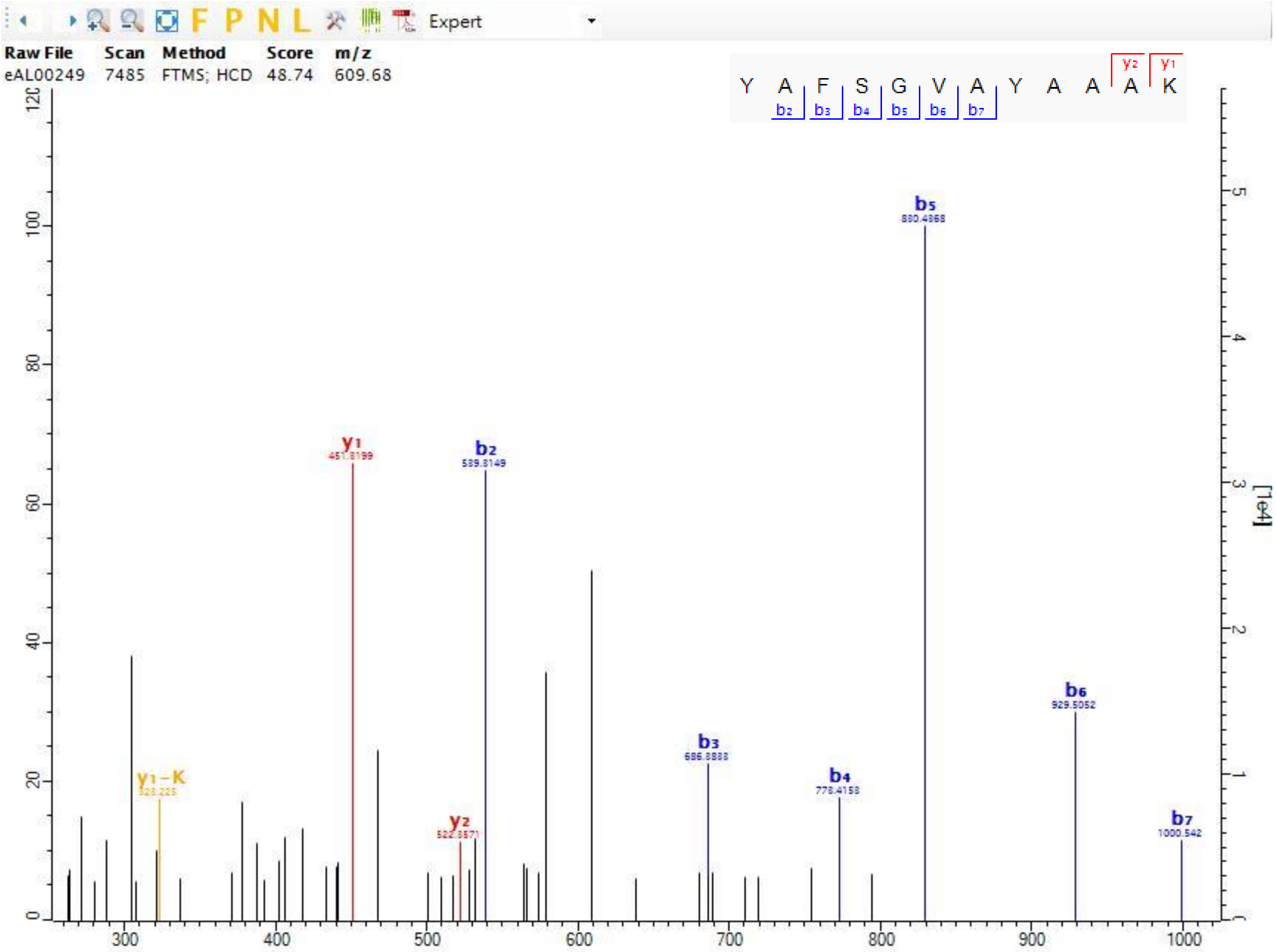

Q96AV8
Transcription factor E2F7

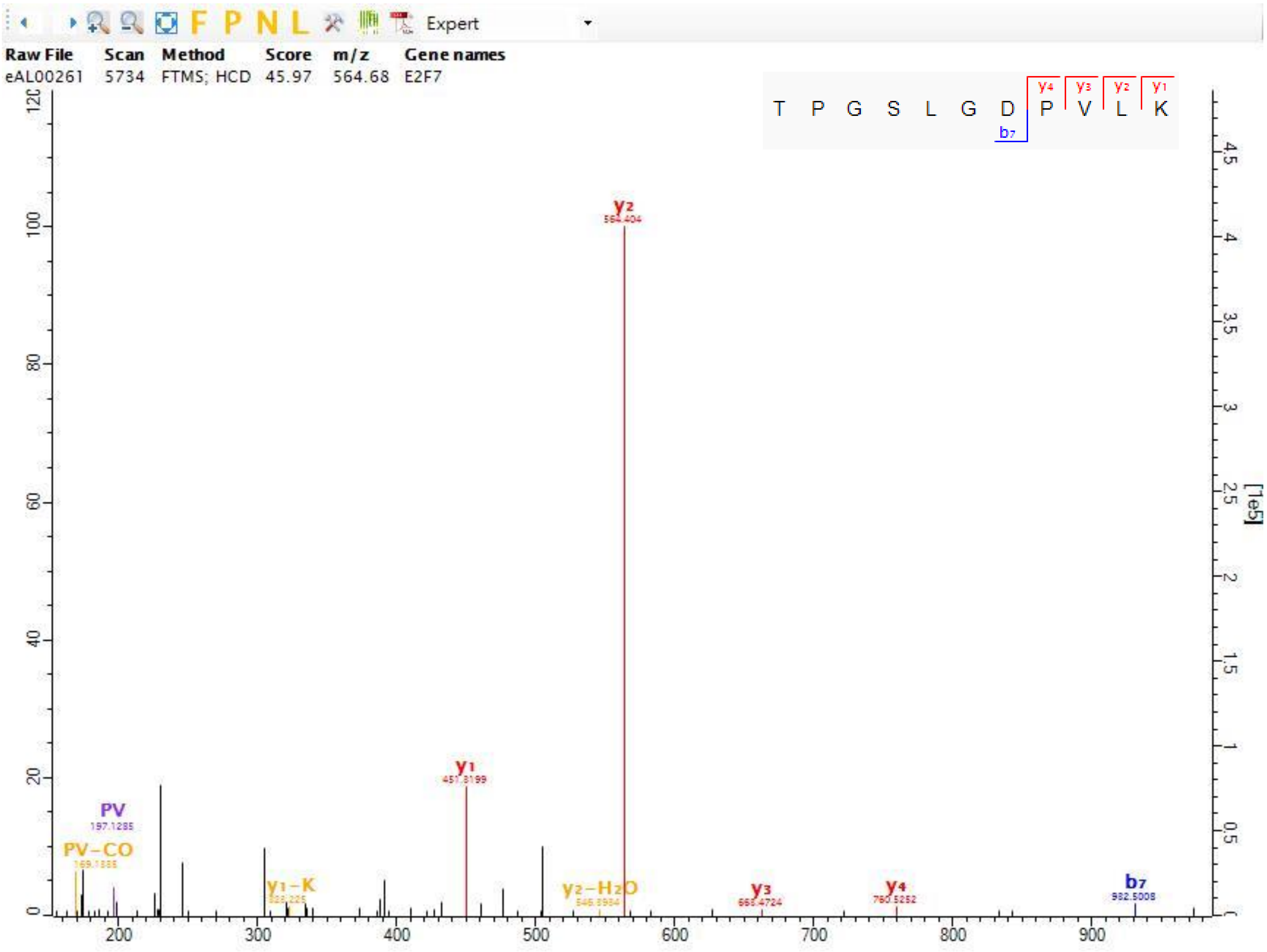

Q9NQV8
PR domain zinc finger protein 8

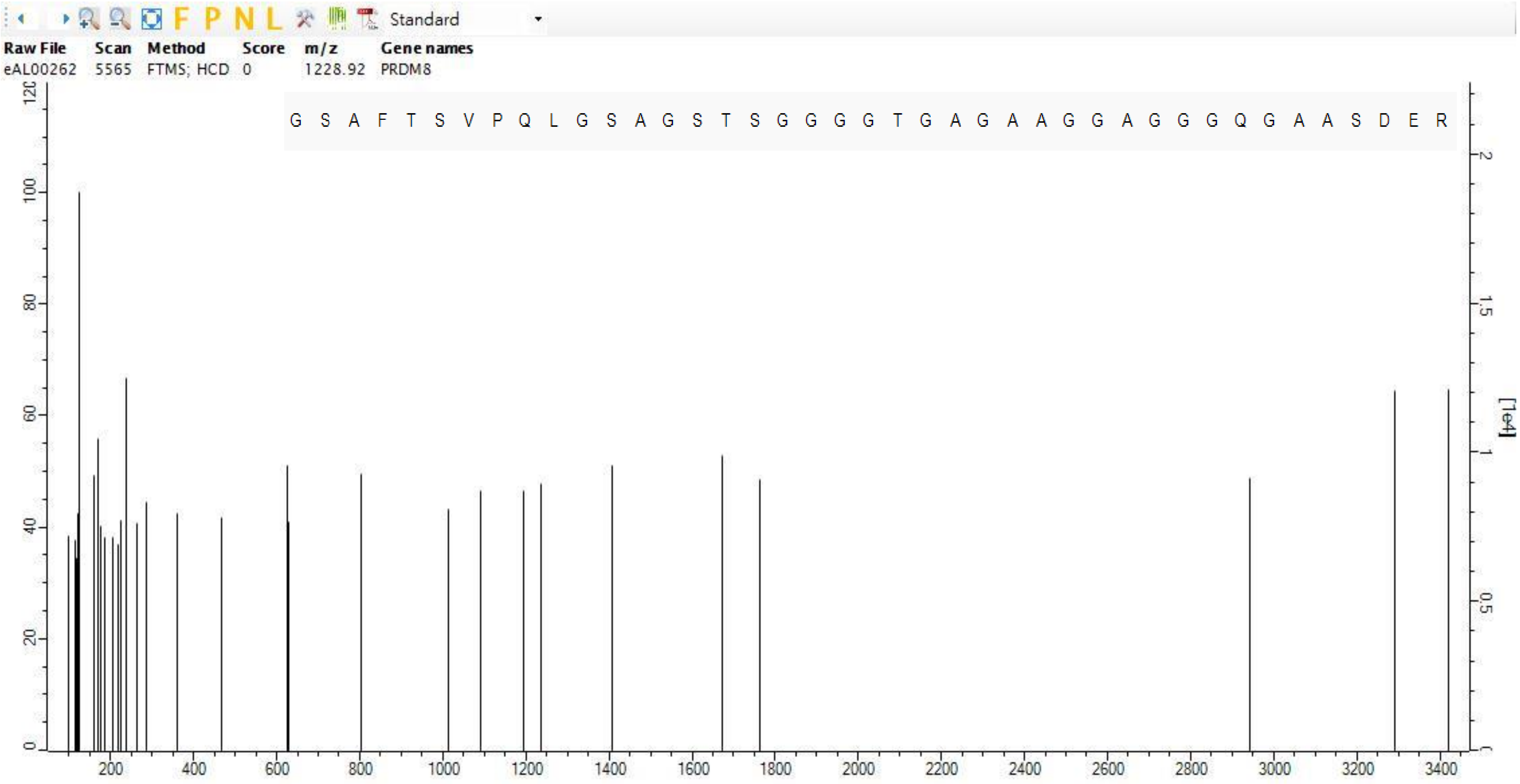

Supplement: Supplemental Figure S1 [file mmc7.pdf]
